# Integrated Metabarcoding and Culturomic-Based Microbiome Profiling of Rice Phyllosphere Reveal Diverse and Functional Bacterial Communities for Blast Disease Suppression

**DOI:** 10.3389/fmicb.2021.780458

**Published:** 2021-11-30

**Authors:** Kuleshwar Prasad Sahu, Asharani Patel, Mukesh Kumar, Neelam Sheoran, Sahil Mehta, Bhaskar Reddy, Pierre Eke, Narayanasamy Prabhakaran, Aundy Kumar

**Affiliations:** ^1^Division of Plant Pathology, ICAR-Indian Agricultural Research Institute, New Delhi, India; ^2^Crop Improvement Group, International Centre for Genetic Engineering and Biotechnology, New Delhi, India

**Keywords:** antibiosis, blast, defense genes, *Magnaporthe oryzae*, microbiome, phyllosphere, rice, immunocompetence

## Abstract

Phyllosphere—the harsh foliar plant part exposed to vagaries of environmental and climatic variables is a unique habitat for microbial communities. In the present work, we profiled the phyllosphere microbiome of the rice plants using 16S rRNA gene amplicon sequencing (hereafter termed metabarcoding) and the conventional microbiological methods (culturomics) to decipher the microbiome assemblage, composition, and their functions such as antibiosis and defense induction against rice blast disease. The blast susceptible rice genotype (PRR78) harbored far more diverse bacterial species (294 species) than the resistant genotype (Pusa1602) that showed 193 species. Our metabarcoding of bacterial communities in phyllomicrobiome revealed the predominance of the phylum, Proteobacteria, and its members *Pantoea*, *Enterobacter*, *Pseudomonas*, and *Erwinia* on the phyllosphere of both rice genotypes. The microbiological culturomic validation of metabarcoding-taxonomic annotation further confirmed the prevalence of 31 bacterial isolates representing 11 genera and 16 species with the maximum abundance of *Pantoea.* The phyllomicrobiome-associated bacterial members displayed antifungal activity on rice blast fungus, *Magnaporthe oryzae*, by volatile and non-volatile metabolites. Upon phyllobacterization of rice cultivar PB1, the bacterial species such as *Enterobacter sacchari*, *Microbacterium testaceum*, *Pantoea ananatis*, *Pantoea dispersa*, *Pantoea vagans*, *Pseudomonas oryzihabitans*, *Rhizobium* sp., and *Sphingomonas* sp. elicited a defense response and contributed to the suppression of blast disease. qRT-PCR-based gene expression analysis indicated over expression of defense-associated genes such as *OsCEBiP*, *OsCERK1*, and phytohormone-associated genes such as *OsPAD4*, *OsEDS1*, *OsPR1.1*, *OsNPR1*, *OsPDF2.2*, and *OsFMO* in phyllobacterized rice seedlings. The phyllosphere bacterial species showing blast suppressive activity on rice were found non-plant pathogenic in tobacco infiltration assay. Our comparative microbiome interrogation of the rice phyllosphere culminated in the isolation and identification of agriculturally significant bacterial communities for blast disease management in rice farming through phyllomicrobiome engineering in the future.

## Introduction

Microbial communities have an evolutionary association with plant populations where they function as metaorganisms in the natural environment. Here, the microbial activities in total termed microbiomes play a pivotal role in plant development and survival (Hartmann et al., 2008; Bulgarelli et al., 2013; Berg et al., 2016). The microbial communities associated with the plants are called plant microbiome and microbial metagenome that often confer functional flexibility to the plant genome (Sessitsch et al., 2012). The plant microbiomes are presumed to modulate a variety of plant functions. However, the ecological role of the phyllosphere microbial communities on plant functional ecology is among the most understudied and underrated aspects in plant biology.

The microbial life on the foliar niche, the phyllosphere microbiome, is constantly exposed to vagaries of weather events, and other agronomic practices in crop husbandry (Lindow and Leveau, 2002). Currently, the epiphytic phyllosphere microbiomes and their natural functions are increasingly investigated in crops like rice, wheat, maize, and soybean (Andrews and Harris, 2000; Bertani et al., 2016; Compant et al., 2019; Sahu et al., 2020). It is further reported that plant-associated bacteria are prolific for the secretion of primary and secondary metabolites and volatiles for plant growth, developmental regulation, and defense against stresses (Munjal et al., 2016; Rascovan et al., 2016; Sheoran et al., 2016; Gómez Expósito et al., 2017; Eke et al., 2019; Ashajyothi et al., 2020; Vandana et al., 2021).

Rice is the primary staple for the nearly three billion world population and contributes to global food security. Rice production is affected by several biotic and abiotic stresses; among them, blast disease caused by ascomycetous fungus *Magnaporthe oryzae* (anamorph *Pyricularia oryzae* Sacc.) is responsible for nearly 30.0% of losses, which can feed 60 million population if prevented preemptively (Dean et al., 2005; Scheuermann et al., 2012; Yasuda et al., 2015; Hashim et al., 2018; Mehta et al., 2019; Prakash et al., 2021). Deployment of fungicides and blast-resistant cultivars are among the blast-combating strategies widely practiced. However, both strategies are under scanner as the chemicals are no longer encouraged due to safety considerations, and the host resistance is not durable (Nalley et al., 2016; Asibi et al., 2019; Sella et al., 2021). Recently, the fungicide residues intercepted on the Indian rice imports have prompted many countries to reject consignments from international trade (Al-Antary et al., 2020). Under this scenario, there is a growing demand among the various stakeholders of the rice production system for an alternative blast mitigation strategy. One promising yet unexplored strategy is the biological control of blast disease by deploying leaf microbiota that shares the same microecological niche with the blast pathogen, *M. oryzae*. So far, the potential of phyllosphere microbial communities sharing the leaf microhabitat with *Magnaporthe*—the incitant of blast disease has not yet been harnessed to mitigate the disease in any crop. Hence, the present investigation was carried out to explore the phyllo-microbiome of rice and exploit them for blast disease suppression by microbiome reengineering.

## Materials and Methods

### Study Location and Sampling for Microbiome Analysis

The 16S rRNA gene amplicon sequencing by NGS (hereafter cited as metabarcoding; Berg et al., 2020), combined with conventional culturomic investigation of phyllomicrobiome, was conducted. For this, we planted rice genotypes in a blast-endemic mountain ecosystem in Palampur, Himachal Pradesh, India (32°6′4.7″N and 76°32′39.79″E) located at an altitude of 1,275 m above mean sea level [weather conditions: mean temperature 22–23°C; precipitation 700–1,000 mm; RH 60.0%; sunshine hours 300–350; source: https://en.climate-data.org; www.worldweatheronline.com]. The rice genotypes were grown during the rice-growing season in August to September 2014. Briefly, rice genotypes PRR78—a blast susceptible variety, and Pusa1602—a near-isogenic line of PRR78 introgressed with *Pi2* gene conferring complete resistance to blast disease (Singh et al., 2012), were planted in parallel rows with a spacing of 20 cm by adopting all crop husbandry practices. Leaf samples were excised at 15 and 30 days post-sowing in sterilized falcon tubes and brought to the laboratory in an insulated cool container maintained at a temperature of 4.0 ±1.0°C and processed for microbiome analysis by metabarcoding and culturomic analysis.

### Profiling of Phyllomicrobiome by Metabarcoding

#### Extraction and Isolation of Epiphytic Microbial Community Genomic DNA

Leaf (5.0 g) samples were shaken with 50 ml of sterile phosphate buffer saline [PBS, g L^–1^ NaCl 8; KCl 0.2; Na_2_HPO_4_ 1.44; KH_2_PO_4_ 0.24; pH-7.4] amended with 0.1% Tween-20 (PBS-T) to dislodge the epiphytic microbiome. The leaf epiphytic microbiome was extracted six times serially in 50 ml of PBS-T by agitating for 30 min at 250 rpm and vortexing for 10 s. Thus, the collected epiphytic–microbial suspension (300 ml) was collected aseptically in a presterilized container and centrifuged at 12,000 × *g* for 60 min at 4.0°C to collect the epiphytic microbial cells. Thus, the obtained pellet was processed to isolate genomic DNA by the cetyltrimethyl ammonium bromide (CTAB) method reported by Moore et al. (2004) with slight modifications like avoidance of phenol in the extraction steps. The quality and quantity of microbial community genomic DNA was determined electrophoretically, spectrophotometrically (Nanodrop 2000, Thermo Scientific, United States), and fluorometrically using Qubit dsDNA BR Assay (Thermo Fisher Scientific Inc., United States).

#### Preparation of Sequencing Libraries for 2 × 300-bp Run Chemistry

The amplicon libraries were prepared using *Nextera XT Index Kit* (Illumina Inc.) as per the 16S rRNA Gene Amplicon Sequencing Library Preparation Protocol (Part no. 15044223 Rev. B). PCR primers for the amplification of the 490-bp hypervariable region of V3–V4 of 16S rRNA gene of Eubacteria were designed, synthesized, and used. The sequences of the primers are V3F: 5′CCTACGGGNGGCWGCAG3′ and V4R: 5′GACTACHVGGGTATCTAATCC3′. The target amplicons were generated using the fusion primer that consists of Illumina adaptors and multiplex index sequence as per the instructions of the manufacturer. The amplicon libraries were purified by 1× AMpure XP beads and checked on Agilent High Sensitivity (HS) chip on Bioanalyzer 2100 and quantified on fluorometer by QubitdsDNA HS-Assay kit (Life Technologies, United States). Quality-passed libraries were equimolar pooled and then sequenced using the IlluminaMiSeq platform with 2 × 300-bp paired-end sequencing chemistry following the protocols of the manufacturer (Illumina, San Diego, CA, United States).

#### Bioinformatic Analyses

Initially, the sequenced raw forward reads (R1) and reverse reads (R2) were scanned and analyzed using the FastQC version (Andrews, 2018) to assess the quality of 16rRNA amplicon reads. Thus, the obtained raw reads were end trimmed and curated using Trimmomatic v0.35 (Bolger et al., 2014) with the following command and settings: (i) remove adapter sequences, (ii) ambiguous reads (reads with unknown nucleotides “N” larger than 5.0%), and (iii) low-quality sequences [reads with more than 10.0% quality threshold (quality value) <20 Phred score]. The resultant quality-passed read pairs were joined using PEAR (Paired-End reAdmergeR) version 0.9.8 (Zhang et al., 2014) with default parameters. The joined paired reads were further processed for downstream taxonomic classification where unpaired reads were discarded. The taxonomic classification of resultant high-quality reads was performed using MG-RAST v4.0, wherein (i) 16S rRNA gene sequence reads were sorted using Sortme RNA, (ii) sorted reads were clustered at ≥97% similarity using CD-HIT method, and (iii) clustered reads were taxonomically classified using SILVA SSU database. The clustered reads and taxon abundance downloaded >100 bases and 90.0% similarity through the best hit classification. Furthermore, PAST v2.17c (Hammer et al., 2001) was used for the determination of α-diversity.

### Phyllosphere Microbiome Interrogation by Culturomic Methods

#### Isolation and Characterization of the Culturable Epiphytic Microbiome

Another set of leaves (500 mg) excised from the rice genotypes were analyzed using the culturomic method on nutrient agar medium [NA, g L^–1^; peptone 5.0; beef extract 3.0; NaCl 5.0; agar 15.0; pH 7.0 ± 0.2]. Briefly, the leaf was agitated with 50 ml of sterile phosphate buffer saline amended with 0.1% Tween-20 (PBST) for 30 min at 250 rpm followed by vortexing for 10 s; the aliquot, thus, obtained was decimally diluted up to 10^–5^. An aliquot of 1.0 ml at 10^–3^, 10^–4^, and 10^–5^ from each sample was pour plated in nutrient agar media supplemented with 2,3,5-tetrazolium chloride (50 mg L^–1^) and incubated at 28 ± 2°C for 72 h for morphotyping the bacterial colonies. The culturable bacterial population and their diversity were assessed and quantified based on morphological traits, such as size, shape, color, texture, and margin. The pure culture of the representative isolates was preserved in −80 and −20°C as glycerol stock (30% V/V) for downstream work.

### Identification of Epiphytic Bacterial Species by 16S rRNA Gene Sequencing

Genomic DNA was isolated by the CTAB method described by Moore et al. (2004) with minor modifications as mentioned previously. Isolated and purified genomic DNA was quantitated and quality analyzed as described above. Finally, the genomic DNA reconstituted at 100 ng μl^–1^ was used as a template in PCR amplification. Box PCR-based DNA fingerprinting was performed for diversity analysis as well as to eliminate the duplicate isolates from the collection (Versalovic et al., 1994); this PCR-based DNA profiling technique specifically amplifies the non-coding conserved sequences in the bacterial genome and is considered a highly discriminatory DNA-fingerprinting technique (Kumar et al., 2004; Eke et al., 2019). Amplicon profiles were resolved in 1.0% agarose gel at 30 V for 10–12 h and imaged (QuantityOne, BioRad, United States). Isolates showing identical amplicon profiles were presumed to be duplicates. PCR amplification of the 16S rRNA gene was performed using primer sets, 27F (27F: 5′-AGAGTTTGATCCTGGCTCAG-3′) and 1492R (1492R: 5′-GGTTACCTTGTTACGACTT-3′), to amplify the 1,465-bp region (Sheoran et al., 2015; Munjal et al., 2016). Then the PCR amplicons resolved in the agarose gel (1.0 %) were purified and eluted using an elution kit according to the instructions of the manufacturer’ (Promega Corporation, United States). The amplicons were sequenced bidirectionally to achieve maximum coverage of the sequences and analyzed using the nucleotide-Basic Local Alignment Search Tool (BLAST) algorithm at the National Center for Biological Information (NCBI); the bacterial species identity was confirmed by closest match. The diversity analysis of culturable phyllosphere bacterial species was calculated using Shannon diversity indices.

### Activity Screening of Epiphytic Phyllospheric Bacteria on *Magnaporthe oryzae*

#### Antifungal Activity *in vitro*

Airborne volatile organic compounds emitted, and diffusible metabolites secreted, during bacterial growth were tested for antifungal activities on *M. oryzae*. Here, we conducted dual-culture confrontation assays, and mycelial inhibition over mock was calculated as described (Sheoran et al., 2015; Munjal et al., 2016). Additionally, the fungicidal or fungistatic nature of the antifungal activity of volatiles on *M. oryzae* was also determined. Here, the volatile exposed mycelia of *M. oryzae* showing complete inhibition were further incubated after replacing bacterial volatile with lid. Depending on the mycelial growth, the bacterial volatile was either categorized as fungicidal or fungistatic on *M. oryzae* (Sahu et al., 2020). The radial mycelial growth of *M. oryzae* was measured, and mycelial inhibition (%) over mock was calculated using the following formula


$$\text{I}=\frac{\text{C-T}}{\text{C}}\times 100$$

where I = percent inhibition

C = colony diameter in control

T = colony diameter in treatment

#### Blast-Suppressive Activity *in planta*

The bacterial isolates showing inhibition of mycelial growth of *M. oryzae* were selected for *in planta* blast control assay. Here, blast-susceptible rice genotype, Pusa Basmati 1, was allowed to germinate in the bacterial cell suspension set at three different bacterial densities (∼10^6^, 10^7^, and 10^8^ CFU ml^–1^) for 5 days. Upon germination, the transplants were further grown in a climate-controlled greenhouse set at a temperature of 28.0 ± 2.0°C, relative humidity of 90.0 ±10.0%, and light/dark cycles of 14/10 h. Thus, the obtained 3-week-old seedlings were foliar sprayed (booster spray) with phyllosphere bacterial suspension (∼10^6^, 10^7^, and 10^8^ CFU ml^–1^) and challenged with the conidia of *M. oryzae-*1637 prepared in water (2.0 × 10^5^ conidia ml^–1^) (Rajashekara et al., 2017). Blast disease severity was determined 7 days post-inoculation on a **0.0–5.0** disease rating scale where **0.0** = no evidence of infection; **1.0** = brown specks <0.5 mm in diameter; **2.0** = brown specks of **0.5–1.0 mm** in diameter; **3.0** = round to elliptical lesions of about **1–3 mm** in diameter; **4.0** = typical spindle-shaped blast lesion of **3.0 mm** or more with little or no coalescence of the lesion; **5.0** = the same as 4.0 but half or more leaves killed by coalescence of lesions. Plants scored **0.0–2.0** were rated resistant, **3.0** as moderately susceptible, and **4.0–5.0** as susceptible (Mackill and Bonman, 1992). The disease severity was calculated using the following formula.


$$\mathrm{Disease}\mathrm{severity}=\frac{\sum \left(\mathrm{Scale}\times \mathrm{Number}\mathrm{of}\mathrm{plants}\mathrm{infected}\right)\times 100}{\mathrm{Total}\mathrm{number}\mathrm{of}\mathrm{plants}\times \mathrm{Maximum}\mathrm{disease}\mathrm{scale}}$$

Furthermore, the percent reduction in disease severity compared with control was estimated using the following formula:


$$\mathrm{Reduction}\mathrm{in}\mathrm{Blast}\mathrm{Severity}=\frac{\text{C}-\text{T}}{\text{C}}\times 100$$

C = disease severity in controlT = disease severity in treatment

#### Phenotyping for Phyllomicrobiome Conferred Immunocompetence

The bacterial isolates showing antifungal activity on *M. oryzae* were selected for the immunocompetence assay. Here, the germination and phenotypic alterations induced on rice seedlings by bacterial isolates were monitored and scored. In this assay, the blast susceptible Pusa Basmati 1 was subjected to germination for 5 days in the presence of bacterial cells at varying densities such as 10^6^, 10^7^, 10^8^, and 10^9^ (CFU mL^–1^). Similarly, seeds germinated in sterile double distilled water served as control. The experiment was performed in three replications with 50 seeds in each replication and repeated twice. The seed germination (%) was calculated using the following formula to evaluate the effect of bacterial interaction on germination.


$$\mathrm{Seed}\mathrm{germination}\left(\%\right)=\frac{\mathrm{Number}\mathrm{of}\mathrm{germinated}\mathrm{seeds}}{\mathrm{Total}\mathrm{number}\mathrm{of}\mathrm{seeds}}\times 100$$

To test the effect of bacterial colonization on the shoot and root growth, five randomly selected seedlings were scored. The percent deviation in shoot and root growth was calculated against the untreated mock seedlings using the following formula. Here, while the negative value indicated growth inhibition, the positive score indicated growth promotion.


$$\text{G}=\frac{\text{T}-\text{C}}{\text{C}}\times 100$$

where G = percent growth of shoot/root

C = length of shoot/root in control

T = length of shoot/root in the treatment

### Assessment of Immunocompetence by qPCR

Having confirmed the blast-suppressive potential of the phyllosphere bacterial species, we performed qPCR experiments to decipher the effect of bacterial supplementation on the expression of genes involved in defense pathways in rice. A total of nine phyllosphere bacterial isolates such as *Pantoea vagans* OsEp-Plm-30B3, *Pantoea ananatis* OsEp-Plm-15B6, *Enterobacter sacchari* OsEp-Plm-15B10, *P. ananatis* OsEp-Plm-30B17, *Pantoea dispersa* OsEp-Plm-15B14, *Rhizobium* sp. OsEp-Plm-30B4, *Microbacterium testaceum* OsEp-Plm-30B1, *Pseudomonas oryzihabitans* OsEp-Plm-15B16, and *Sphingomonas* sp. OsEp-Plm-15B2 that showed blast-suppressive activity on rice was chosen for the study.

Briefly, whole seedlings of Pusa Basmati 1, bacterized with 2 × 10^7^ CFU m^–1^ and sampled at 24-h interval for three consecutive days were immediately snap frozen in liquid nitrogen to stop all the cellular metabolic activity and then stored instantly at −80°C until further use. The total RNA was isolated using the SV Tool RNA isolation system according to the instructions of the manufacturer (Promega, Madison, WI, United States). The quality and quantity of RNA were assessed spectrophotometrically (NanoDrop 2000, Thermo Scientific, United States) and electrophoretically.

### Transcriptional Analysis of Genes Associated With Immunocompetence

Eight rice genes, such as *OsCEBiP* (Akamatsu et al., 2013), *OsCERK1* (Kouzai et al., 2014), *OsPAD4* (Ke et al., 2014), *OsEDS1* (Ke et al., 2019), *OsNPR1* (Sugano et al., 2010), *OsPDF2.2* (Thomma et al., 2002), *Os*FMO1 (Koch et al., 2006; Mishina and Zeier, 2006), and *Os*PR1.1 (Breen et al., 2017), which were reported to play a role in rice defense, were selected; PCR primers targeting the above defense genes are furnished in Supplementary Tables 1, 2. qPCR was performed in Real-Time Thermal Cycler (LightCycler 96, Roche Life Science, Switzerland) using GoTaq^®^ 1-Step RT-qPCR System (Promega Corporation, United States); qPCR reaction conditions were as follows: one cycle of reverse transcription at 37°C for 15 min followed by reverse transcriptase inactivation step at 95°C for 10 min followed by 30 cycles at 95°C for 10 s, annealing at 58°C for 30 s and extension at 72°C for 30 s followed by three-step melting at 95°C for 10 s, 63°C for 60 s, and 97°C for 1.0 s, and then a final cooling at 37°C for 30 s. Later, cyclic threshold data points were analyzed for the determination of gene expression relative to the reference housekeeping *OsActin* gene using the software LightCycler^®^96 Roche. The mean *Ct* values were considered for the calculation of 2^–ΔΔ*CT*^ to estimate the fold changes in gene expression.

### Hypersensitive Reaction on Tobacco

Upon bacterial infiltration on tobacco leaves, potential plant pathogenic bacteria are known to induce hypersensitive reactions (HR) (Klement, 1963); this is considered as a test to ascertain the plant pathogenic nature of bacterial isolates. The best performing nine bacterial isolates for suppression of blast disease were selected for this assay. Tobacco (*Nicotiana tabacum*) plants were grown under greenhouse conditions at 20°C, 50–60% relative humidity, and 12/12-h light/dark per day. Fully expanded leaves of 2 to 3-month-old plantlets were used in all experiments. Bacterial inoculum (1.0 × 10^8^ CFU ml^–1^, absorbance at 600 nm = 1.0 OD) was infiltrated onto the leaves using a sterile hypodermal syringe. Thus, treated plants were incubated at 25–30°C under greenhouse conditions (12/12 h of dark/light photoperiods). Similarly, leaves infiltrated with sterile distilled water alone served as a negative control, and a well-known bacterial pathogen, *Ralstonia solanacearum*, served as a positive control. Plant responses to the bacterial infiltration on tobacco leaves were recorded after 24-h post-inoculation.

### Statistical Analysis

All datasets were analyzed using the data analytical tool available in MS Office Excel 2013. The analyzed data obtained were subjected to significance testing by analysis of variance (ANOVA) at a *p* ≤ 0.05 level of significance. Furthermore, various parameters like the standard error of the mean (SEm), standard error of the difference between two means (SEd), critical difference (CD), and coefficient of variation (CV) were estimated. For figures and tables, the values are represented as the mean of all biological and technical replicates. For the qPCR data analysis, the fold change values calculated for the defense genes were imported into the GraphPad Prism program (https://www.graphpad.com/scientific-software/prism), and two-way ANOVA was conducted using Bonferroni *post-hoc* test for determining the statistical significance at *p ≤ 0.05, ^**^p = 0.001, and ^***^p = 0.0001.

## Results

### 16S rRNA Barcode Sequence Read Statistics and Diversity Indices

Phyllomicrobiome profiling of blast-susceptible (PRR78) and blast-resistant rice (Pusa1602) genotypes planted in blast-endemic locations was conducted using integrated 16S rRNA gene amplicon sequencing and microbiological methods. The total curated sequence generated is in the range of 4, 31,222 for Pusa1602 and 1, 81,250 for PRR78 reads (Table 1). The α-diversity indices (Shannon diversity) were 1.25 for Pusa1602 and 1.85 for PRR78. Other diversity indices were also marginally higher for PRR78 than Pusa1602 revealing that the blast-susceptible genotype harbored more diverse microbial species than the resistant type (Table 1).

**TABLE 1:** Metabarcoding statistics and diversity indices of phyllosphere microbiome.

Parameters	Sample origin: Mid Himalayan mountain—Palampur, India	
	
	Pusa1602	PRR78
MG-RAST accession number*	mgm4619774.3	mgm4621255.3
Number of base pairs	201,387,096	221,346,634
Total number of sequences	4,39,681	4,68,994
Total number of reads	431,222	181,250
Simpson	0.6304	0.6936
Shannon	1.253	1.851
Evenness	0.01813	0.02166
Fisher-α	19.03	33.71
Berger–Parker	0.4339	0.5018
Chao-1	310.4	421.7
Observed species	193	294

*https://www.mg-rast.org/.

### Structure and Composition of Phyllomicrobiome on Blast-Susceptible and Resistant Rice Genotypes

**FIGURE 1:**
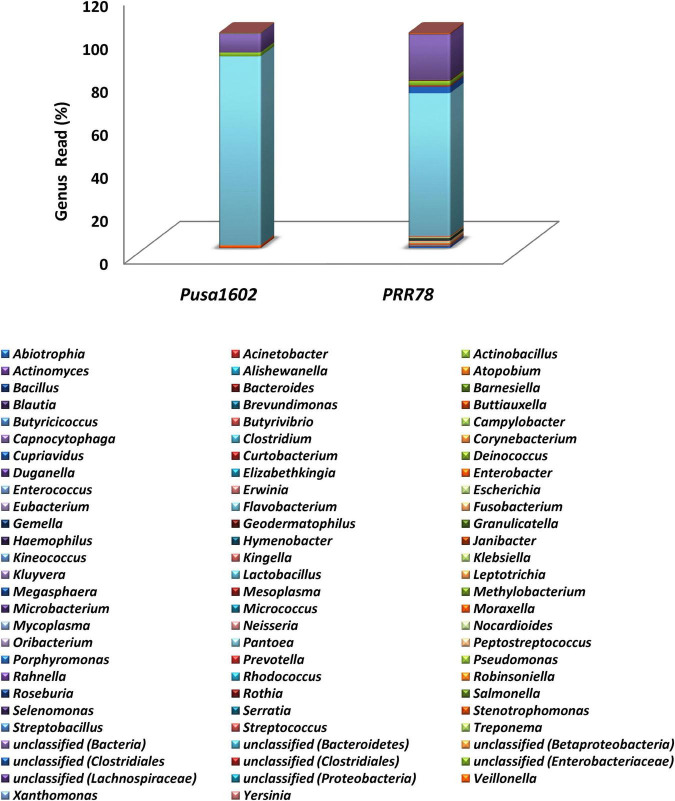
Genus-level relative abundance of phyllosphere bacterial communities on rice genotypes; refer to Supplementary Figure 1 for other taxonomic hierarchy.

The metabarcoding-assisted taxonomic profiling of the phyllosphere microbiome of two rice genotypes revealed the abundance of bacterial phyla, Proteobacteria (70.7–91.0%), and class Gamma Proteobacteria on both the genotypes (70.4–91.0%). Bacterial communities belong to the order *Enterobacteriales* (89.2%) followed by *Pseudomonadales* (1.7%) was found dominant on Pusa1602, while *Enterobacteriales* (68.0%) followed by *Bacteroidales* (3.7%) and *Pseudomonadales* (2.2%) were in high frequency on susceptible genotype, PRR78. Further at the family level, *Enterobacteriaceae* (89.2%) followed by *Pseudomonadaceae* (1.7%) in the resistant genotype and *Enterobacteriaceae* (68.0%) followed by *Porphyromonadaceae* (3.0%) and *Pseudomonadaceae* (2.0%) in the susceptible genotype were found overrepresented. Bacterial genus *Pantoea* (67.3–87.7%) was the most dominant bacteria on both the genotypes. Other dominant genera are *Pseudomonas*, *Enterobacter*, *Buttiauxella*, and *Erwinia* in the resistant genotype and *Porphyromonas*, *Pseudomonas*, *Abiotrophia*, *Enterobacter*, and *Gemella* in the susceptible genotype (Figure 1, Supplementary Figure 1, and Supplementary Table 3).

### Validation of Metabarcoding Sequence Data by Culturomic Methods

#### Enumeration of Cultivable Microbiome and Identification of Bacterial Communities

**FIGURE 2:**
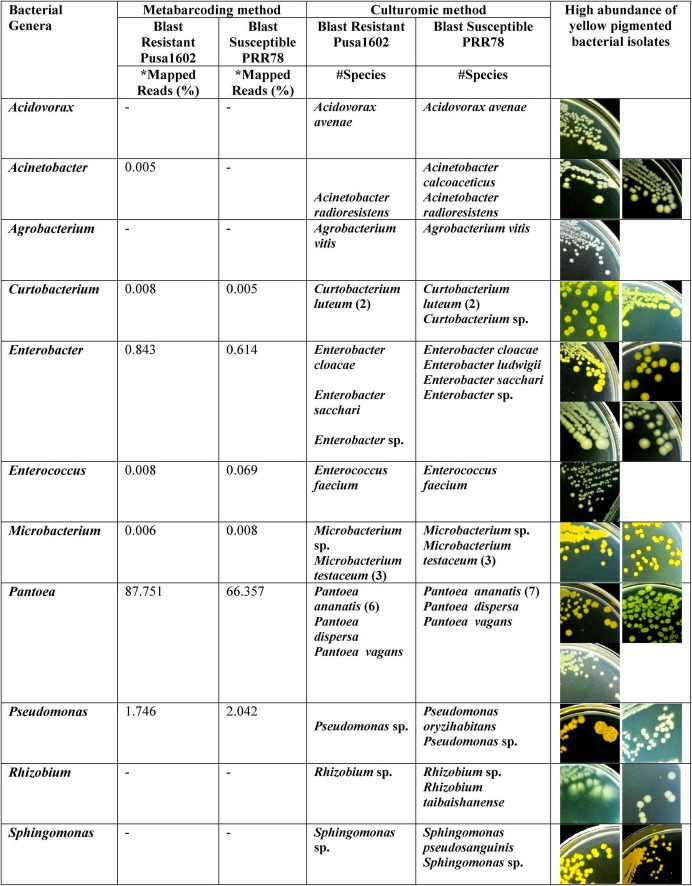
Microbiological culturomic validation of bacterial species composition in phyllomicrobiome; bacterial species belonging to yellow-pigmented *Curtobacterium*, *Enterobacter*, *Microbacterium*, *Pseudomonas*, *Pantoea*, and *Sphingomonas* were found dominant on the phyllosphere. Data in parentheses represent the number of isolates cultured. *Species identity in Silva Database. #Species identity in GenBank database.

Both the genotypes recorded the nearly identical epiphytic bacterial population (1.55–5.6 log CFU g^–1^) (Supplementary Table 4). A total of 37 distinct morphotypes of bacterial isolates were enumerated with a diversity index ranging from 1.48 to 1.82 for all the cultured phyllosphere microbiome. The 30-day-old phyllosphere showed more bacterial population (21 morphotypes) and diversity compared with the 15-day seedlings (16 morphotypes). The results of diversity indices further revealed more bacterial diversity in the susceptible genotypes than in the resistant genotypes. The diversity indices of epiphytic bacteria on the rice phyllosphere are presented in Supplementary Table 5. BOX-PCR DNA fingerprinting of all 37 morphotypes culminated in 31 distinct BOX Amplicon Groups (Supplementary Figure 2). Isolates with identical amplicon profiles were considered duplicates, and a representative isolate for each of the BOX groups was retained and subjected to downstream work. The bacterial species identity was established through a 16S rRNA gene sequence analysis. The 31 distinct BOX amplicon groups represented 11 genera and 16 species. We also observed high-frequency occurrence of bacterial species like *Acinetobacter* (2), *Curtobacterium* (3), *Enterobacter* (4), *Microbacterium* (4), *Pantoea* (9), *Pseudomonas* (2), *Rhizobium* (2), and *Sphingomonas* (2) on the rice phyllosphere (Table 2 and Supplementary Figures 3, 4a–k). All cultured bacterial genera were also found among the mapped reads in the metabarcoding analysis (Figure 2 and Table 3).

**TABLE 2:** Identification of cultivated phyllosphere bacterial isolates by 16S rRNA gene sequencing.

Sequence ID	Organism	Sequence length (bp)	*Host	GenBank accession
OsEp_Plm_15B9	*Acinetobacter calcoaceticus*	1,409	PRR78	MT367784
OsEp_Plm_15B13	*Curtobacterium* sp.	1,400	PRR78	MT367788
OsEp_Plm_30B20	*Enterobacter ludwigii*	1,422	PRR78	MT367806
OsEp_Plm_30B8	*Pantoea ananatis*	1,401	PRR78	MT367799
OsEp_Plm_15B16	*Pseudomonas oryzihabitans*	1,403	PRR78	MT367791
OsEp_Plm_15B8	*Rhizobium taibaishanense*	1,349	PRR78	MT367783
OsEp_Plm_30B9	*Sphingomonas pseudosanguinis*	1,400	PRR78	MT367800
OsEp_Plm_15B4	*Acidovorax avenae*	1,400	PRR78 and Pusa1602	MT367779
OsEp_Plm_15B15	*Acinetobacter radioresistens*	1,402	PRR78 and Pusa1602	MT367790
OsEp_Plm_30B7	*Agrobacterium vitis*	1,400	PRR78 and Pusa1602	MT367798
OsEp_Plm_15B3	*Curtobacterium luteum*	1,391	PRR78 and Pusa1602	MT367778
OsEp_Plm_15B12	*Curtobacterium luteum*	1,393	PRR78 and Pusa1602	MT367787
OsEp_Plm_30B10	*Enterobacter cloacae*	1,403	PRR78 and Pusa1602	MT367801
OsEp_Plm_15B10	*Enterobacter sacchari*	1,419	PRR78 and Pusa1602	MT367785
OsEp_Plm_15B11	*Enterobacter* sp.	1,402	PRR78 and Pusa1602	MT367786
OsEp_Plm_15B7	*Enterococcus faecium*	1,409	PRR78 and Pusa1602	MT367782
OsEp_Plm_15B5	*Microbacterium* sp.	1,379	PRR78 and Pusa1602	MT367780
OsEp_Plm_15B1	*Microbacterium testaceum*	1,400	PRR78 and Pusa1602	MT367776
OsEp_Plm_30B1	*Microbacterium testaceum*	1,398	PRR78 and Pusa1602	MT367792
OsEp_Plm_30B5	*Microbacterium testaceum*	1,401	PRR78 and Pusa1602	MT367796
OsEp_Plm_15B6	*Pantoea ananatis*	1,405	PRR78 and Pusa1602	MT367781
OsEp_Plm_30B2	*Pantoea ananatis*	1,404	PRR78 and Pusa1602	MT367793
OsEp_Plm_30B6	*Pantoea ananatis*	1,408	PRR78 and Pusa1602	MT367797
OsEp_Plm_30B15	*Pantoea ananatis*	1,412	PRR78 and Pusa1602	MT367803
OsEp_Plm_30B17	*Pantoea ananatis*	1,384	PRR78 and Pusa1602	MT367804
OsEp_Plm_30B19	*Pantoea ananatis*	1,417	PRR78 and Pusa1602	MT367805
OsEp_Plm_15B14	*Pantoea dispersa*	1,410	PRR78 and Pusa1602	MT367789
OsEp_Plm_30B3	*Pantoea vagans*	1,409	PRR78 and Pusa1602	MT367794
OsEp_Plm_30B14	*Pseudomonas* sp.	1,408	PRR78 and Pusa1602	MT367802
OsEp_Plm_30B4	*Rhizobium* sp.	1,360	PRR78 and Pusa1602	MT367795
OsEp_Plm_15B2	*Sphingomonas* sp.	1,373	PRR78 and Pusa1602	MT367777

*Isolated from rice leaf excised from PRR78 and Pusa1602 planted in Palampur, Himachal Pradesh, India.

**TABLE 3:** Quantification and identification of bacterial population on rice phyllomicrobiome by integrated metabarcoding and culturomic methods.

Genus	Metabarcoding method				Culturomic method	
	Blast-resistant Pusa1602		Blast-susceptible PRR78		Blast-resistant Pusa1602	Blast-susceptible PRR78
	*Read count	Reads (%)	*Read count	Reads (%)	Log CFU g^–1^	Log CFU g^–1^
*Pantoea*	423,893	87.751	137,297	66.357	4.73	5.17
*Pseudomonas*	8,433	1.746	4,226	2.042	3.23	4.47
*Enterobacter*	4,071	0.843	1,270	0.614	4.13	5.00
*Buttiauxella*	1,210	0.250	−	−	−	−
*Erwinia*	501	0.104	264	0.128	−	−
*Klebsiella*	402	0.083	769	0.372	−	−
*Clostridium*	144	0.030	172	0.083	−	−
*Salmonella*	141	0.029	97	0.047		
*Bacteroides*	116	0.024	346	0.167	−	−
*Enterococcus*	40	0.008	142	0.069	3.65	2.16
*Curtobacterium*	38	0.008	10	0.005	2.24	4.64
*Kineococcus*	35	0.007	139	0.067	−	−
*Microbacterium*	27	0.006	16	0.008	5.66	5.90
*Acinetobacter*	22	0.005	−	−	3.49	3.74
*Abiotrophia*	−	−	1,522	0.736	−	−
*Acidovorax*	−	−	−	−	4.54	5.37
*Agrobacterium*	−	−	−	−	3.87	2.47
*Butyrivibrio*	−	−	134	0.065	−	−
*Campylobacter*	−	−	101	0.049	−	−
*Capnocytophaga*	−	−	518	0.250	−	−
*Elizabethkingia*	−	−	206	0.100	−	−
*Escherichia*	−	−	755	0.365	−	−
*Flavobacterium*	−	−	152	0.073	−	−
*Fusobacterium*	−	−	1,093	0.528	−	−
*Gemella*	−	−	1,213	0.586	−	−
*Granulicatella*	−	−	807	0.390	−	−
*Haemophilus*	−	−	475	0.230	−	−
*Kluyvera*	−	−	135	0.065	−	−
*Leptotrichia*	−	−	220	0.106	−	−
*Moraxella*	−	−	382	0.185	−	−
*Neisseria*	−	−	450	0.217	−	−
*Porphyromonas*	−	−	6,355	3.071	−	−
*Prevotella*	−	−	1,064	0.514	−	−
*Rhizobium*	−	−	−	−	5.13	5.43
*Rothia*	−	−	696	0.336		
*Sphingomonas*	−	−	−	−	4.17	4.82
*Streptobacillus*	−	−	107	0.052	−	−
*Veillonella*	−	−	608	0.294	−	−

*Genus with less than 10 reads were not considered.

### Activity Screening Against Rice Blast Fungus *Magnaporthe oryzae*

#### Screening for Antifungal Activity

Dual-plate confrontation assay showed inhibition of the mycelial growth of *M. oryzae* by both volatiles and secreted metabolites produced by bacterial species. Among the 31 bacteria evaluated, 11 phyllosphere-associated bacterial isolates displayed over 40.0% inhibition of mycelial growth by their secreted metabolites (Table 4 and Supplementary Figure 5). The antagonistic bacterial isolates represented species, such as *Acinetobacter calcoaceticus*, *Enterobacter cloacae*, *E. sacchari*, *P. ananatis*, *P. vagans*, *P. oryzihabitans*, and *Sphingomonas* sp. Similarly, a total of 12 of them completely inhibited the growth of *M. oryzae* (100% inhibition) by bacterial volatile organic compounds (Table 4 and Supplementary Figure 6). The antifungal volatile emitting bacterial isolates represented the species, such as *E. sacchari*, *M. testaceum*, *P. ananatis*, *P. dispersa*, *P. vagans*, *P. oryzihabitans*, and *Rhizobium* sp. Furthermore, while the volatile of seven bacterial isolates displayed fungicidal activity, the other five bacteria released fungistatic volatiles against *M. oryzae* as mycelial growth re-emerged upon removal of the volatile exposure (Supplementary Figure 7 and Supplementary Table 6).

**TABLE 4:** Antifungal activity of phyllosphere bacteria by secreted metabolites and volatile compounds against *Magnaporthe oryzae*.

Bacterial isolates	Mycelial inhibition (%)	
	Volatile compounds	Secretory compounds
1. *Enterobacter sacchari* OsEp-Plm-15B10	100.0	57.4
2. *Microbacterium testaceum* OsEp-Plm-30B1	100.0	38.9
3. *Pantoea dispersa* OsEp-Plm-15B14	100.0	35.2
4. *Pantoea ananatis* OsEp-Plm-15B6	100.0	19.4
5. *Pantoea ananatis* OsEp-Plm-30B2	100.0	53.7
6. *Pantoea vagans* OsEp-Plm-30B3	100.0	47.2
7. *Pantoea ananatis* OsEp-Plm-30B6	100.0	45.4
8. *Pantoea ananatis* OsEp-Plm-30B8	100.0	7.4
9. *Pantoea ananatis* OsEp-Plm-30B17	100.0	52.8
10. *Pantoea ananatis* OsEp-Plm-30B19	100.0	8.3
11. *Pseudomonas oryzihabitans* OsEp-Plm-15B16	100.0	41.7
12. *Rhizobium* sp. OsEp-Plm-30B4	100.0	5.6
13. *Sphingomonas* sp. OsEp-Plm-15B2	62.9	53.7
14. *Agrobacterium vitis* OsEp-Plm-30B7	77.9	15.7
15. *Enterobacter cloacae* OsEp-Plm-30B10	76.4	46.3
16. *Pantoea ananatis* OsEp-Plm-30B15	57.9	13.9
17. *Microbacterium testaceum* OsEp-Plm-30B5	57.1	4.6
18. *Enterobacter ludwigii* OsEp-Plm-30B20	56.4	4.6
19. *Curtobacterium luteum* OsEp-Plm-15B12	48.6	8.3
20. *Acidovorax avenae* OsEp-Plm-15B4	45.7	6.5
21. *Acinetobacter radioresistens* OsEp-Plm-15B15	45.7	8.3
22. *Microbacterium testaceum* OsEp-Plm-15B1	42.9	7.4
23. *Curtobacterium* sp. OsEp-Plm-15B13	38.6	7.4
24. *Acinetobacter calcoaceticus* OsEp-Plm-15B9	37.9	44.4
25. *Enterococcus faecium* OsEp-Plm-15B7	35.0	23.2
26. *Sphingomonas pseudosanguinis* OsEp-Plm-30B9	35.0	6.5
27. *Microbacterium* sp. OsEp-Plm-15B5	34.3	8.3
28. *Rhizobium taibaishanense* OsEp-Plm-15B8	32.9	8.3
29. *Enterobacter* sp. OsEp-Plm-15B11	32.1	39.8
30. *Pseudomonas* sp. OsEp-Plm-30B14	31.4	3.7
31. *Curtobacterium luteum* OsEp-Plm-15B3	29.3	11.1
Mock	0.0	0.0
C.D.	12.0	4.8
SE (m)	4.3	1.7
SE (d)	6.1	2.4
C.V. (%)	13.2	12.8
F (calc.)	52.1	130.5
F (tab.)	1. 6	1.6

#### Suppressive Effect of Phyllomicrobiome on Blast Disease

**FIGURE 3:**
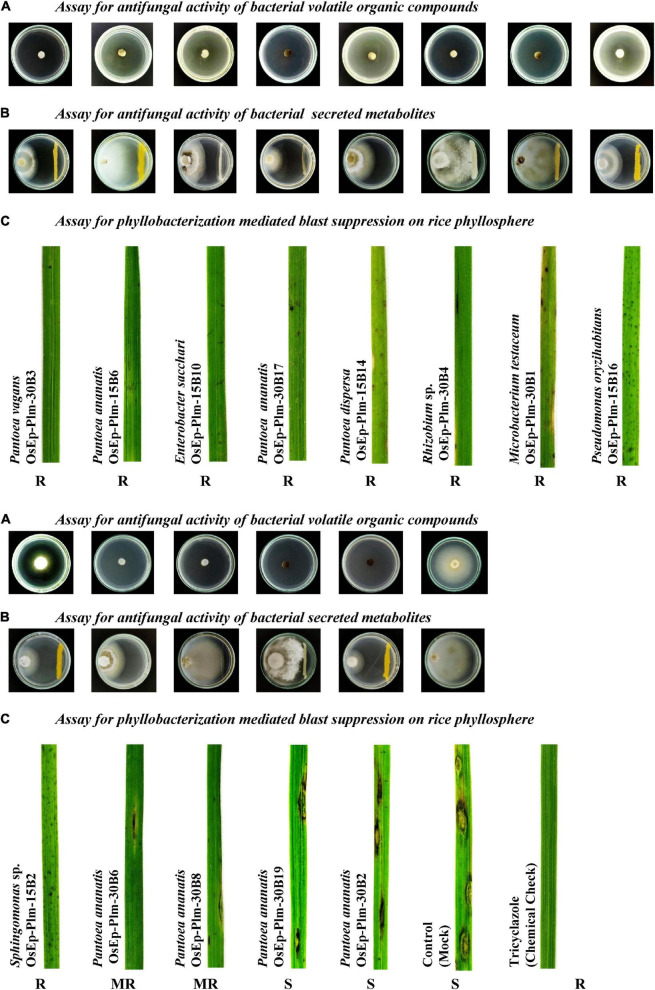
Suppressive effects of phyllosphere bacterial inoculation on *Magnaporthe oryzae* and the rice blast disease. **(A)** Phyllosphere bacteria displayed volatile mediated antifungal activity on *Magnaporthe oryzae*. **(B)** A few isolates showed secreted metabolite mediated antifungal activity on *Magnaporthe oryzae*. **(C)** Nine of the tested 13 bacteria isolates showed blast-suppressive activity on rice leaf.

Blast-susceptible rice cultivar, Pusa Basmati 1, was used for evaluating the blast-suppressive effects of rice phyllomicrobiome. A total of 13 bacterial isolates representing *Pantoea* (eight strains), *Enterobacter* (one), *Microbacterium* (one), *Pseudomonas* (one), *Rhizobium* (one), and *Sphingomonas* (one) were evaluated at three different cell densities. Blast incidence and severity were scored as per the blast score chart recommended by Mackill and Bonman (1992). Most of the bacterial isolates were found to reduce the blast disease development in the plants of the susceptible rice cultivar at all tested doses. Maximum reduction in disease severity was shown by *P. vagans* OsEp-Plm-30B3 (81.9%), *P. ananatis* OsEp-Plm-15B6 (81.5%), *E. sacchari* OsEp-Plm-15B10 (78.1%), *P. ananatis* OsEp-Plm-30B17 (77.7%), *P. dispersa* OsEp-Plm-15B14 (76.2%), *Rhizobium* sp. OsEp-Plm-30B4 (69.8%), *M. testaceum* OsEp-Plm-30B1 (67.5%), *P. oryzihabitans* OsEp-Plm-15B16 (52.4%), and *Sphingomonas* sp. OsEp-Plm-15B2 (51.8%) (Figure 3 and Table 5). Nine of the 13 bacterial isolates showed over 50% reduction of blast severity in all bacterial titers. Interestingly, the reduction in blast severity could not be correlated with the bacterial cell densities used for phyllobacterization.

**TABLE 5:** Suppressive effects of phyllobacterization on rice blast disease.

Bacterial species/isolate	Bacterial dose (0.01 OD at A600 nm = ∼10^6^ cfu per ml)		Bacterial dose (0.1 OD at A600 nm = ∼10^7^cfu per ml)		Bacterial dose (1.0 OD at A600 nm = ∼10^8^ cfu per ml)		Mean	
				
	*Blast severity	**Reduction in severity (%)	*Blast severity	**Reduction in severity (%)	*Blast severity	**Reduction in severity (%)	*Blast severity	**Reduction in severity (%)
***Pantoea vagans* OsEp-Plm-30B3**	11.3	77.9	7.0	86.2	9.4	81.5	9.2	81.9
***Pantoea ananatis* OsEp-Plm-15B6**	8.1	84.0	13.3	73.8	6.8	86.7	9.4	81.5
***Enterobacter sacchari* OsEp-Plm-15B10**	11.7	76.9	13.0	74.5	8.7	83.0	11.1	78.1
***Pantoea ananatis* OsEp-Plm-30B17**	15.8	68.9	12.1	76.3	6.2	87.9	11.3	77.7
***Pantoea dispersa* OsEp-Plm-15B14**	8.0	84.3	15.5	69.5	12.8	74.9	12.1	76.2
***Rhizobium* sp. OsEp-Plm-30B4**	13.4	73.7	18.3	63.9	14.3	71.9	15.3	69.8
***Microbacterium testaceum* OsEp-Plm-30B1**	19.0	62.6	11.4	77.5	19.1	62.5	16.5	67.5
***Pseudomonas oryzihabitans* OsEp-Plm-15B16**	22.4	55.9	21.9	56.9	28.3	44.4	24.2	52.4
***Sphingomonas* sp. OsEp-Plm-15B2**	20.9	58.9	24.2	52.5	28.5	43.9	24.5	51.8
***Pantoea ananatis* OsEp-Plm-30B6**	21.5	57.7	28.9	43.2	34.8	31.6	28.4	44.2
***Pantoea ananatis* OsEp-Plm-30B8**	29.5	41.9	26.9	47.1	32.2	36.6	29.6	41.9
***Pantoea ananatis* OsEp-Plm-30B19**	41.7	18.0	42.2	17.0	35.0	31.1	39.6	22.0
***Pantoea ananatis* OsEp-Plm-30B2**	54.6	−7.5	42.9	15.7	39.0	23.2	45.5	10.5
**Control**	50.8	0.0	50.8	0.0	50.8	0.0	50.8	0.0
**Tricyclazole**	6.8	86.7	10.1	80.1	8.1	84.0	8.33	83.6

*Disease severity was calculated using the following formula:
$$\mathrm{Disease}\mathrm{severity}=\frac{\sum \left(\mathrm{Scale}\times \mathrm{Number}\mathrm{of}\mathrm{plants}\mathrm{infected}\right)\times 100}{\mathrm{Total}\mathrm{number}\mathrm{of}\mathrm{plants}\times \mathrm{Maximum}\mathrm{disease}\mathrm{scale}}$$

**Blast severity reduction was estimated using the following formula:
$$\mathrm{Blast}\mathrm{severity}\mathrm{reduction}=\frac{\text{C}-\text{T}}{\text{C}}\times 100$$

*C = disease severity in control.*

*T = disease severity in treatment.*

### Phenotyping for Phyllomicrobiome Conferred Immunocompetence

**FIGURE 4:**
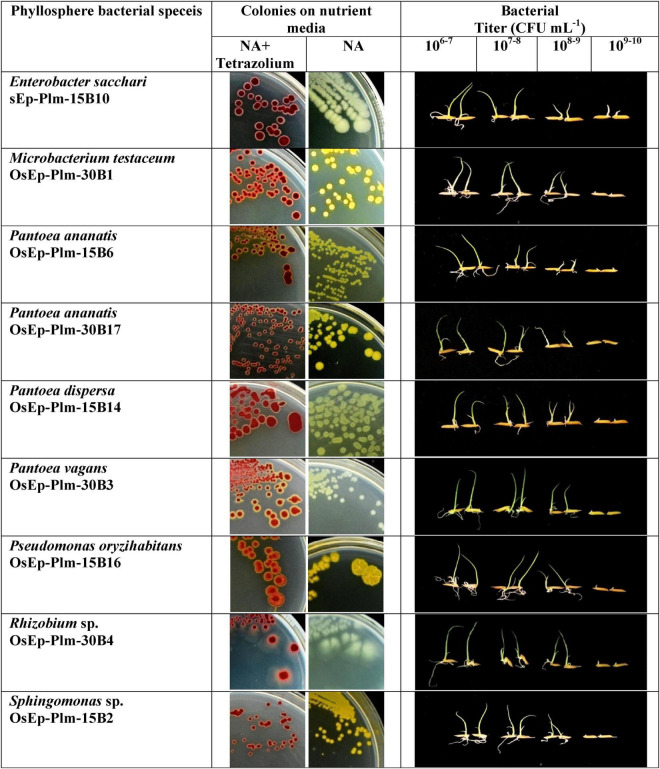
Assay for *phyllomicrobiome-conferred immunocompetence.* Seedling growth inhibition as a phenotypic marker of microbiome-conferred immunocompetence, observed with nine blast-suppressive bacterial isolates, represented six genera such as *Enterobacter*, *Microbacterium*, *Pantoea*, *Pseudomonas*, *Rhizobium*, and *Sphingomonas*. The rice phyllosphere bacteria showed various shades of yellow pigmentation; the pink color appearance of the bacterial colony is due to the reduction of tetrazolium dye into insoluble formazan; the inhibition of shoot and root growth—an indicator of innate immunity, can be seen in plantlets interacting with high bacterial titer.

An experiment was conducted to study the potential of phyllosphere bacteria to confer immunocompetence in rice. Seedling growth inhibition due to microbial interactions is touted as a phenotypic marker for the induced immune response in plants. Here, the seed germination and the seedling emergence were monitored during bacterial interaction that showed germination in the range of 40.0–86.7% across various bacterial inoculations. Maximum germination was observed in *E. sacchari* OsEp-Plm-15B10, while the minimum germination was observed in *P. ananatis* OsEp-Plm-30B2 (Supplementary Figure 8 and Supplementary Table 7). However, the germinability of rice seeds reduced upon increasing bacterial density with poor germination observed at a titer of 10^9^ CFU ml^–1^. The bacterial inoculation on seeds and seedlings appeared to trigger the plant phenotypic alteration especially on the shoot and root growth. All nine bacterial isolates representing six genera induced a density-dependent alteration on the shoot and root emergence and growth (Supplementary Tables 8, 9). In particular, the shoot and root growth of rice was found inhibited at high bacterial titer (10^9^ CFU ml^–1^) (Figure 4).

### qPCR Assay on Phyllobacterization-Mediated Immunocompetence

**FIGURE 5:**
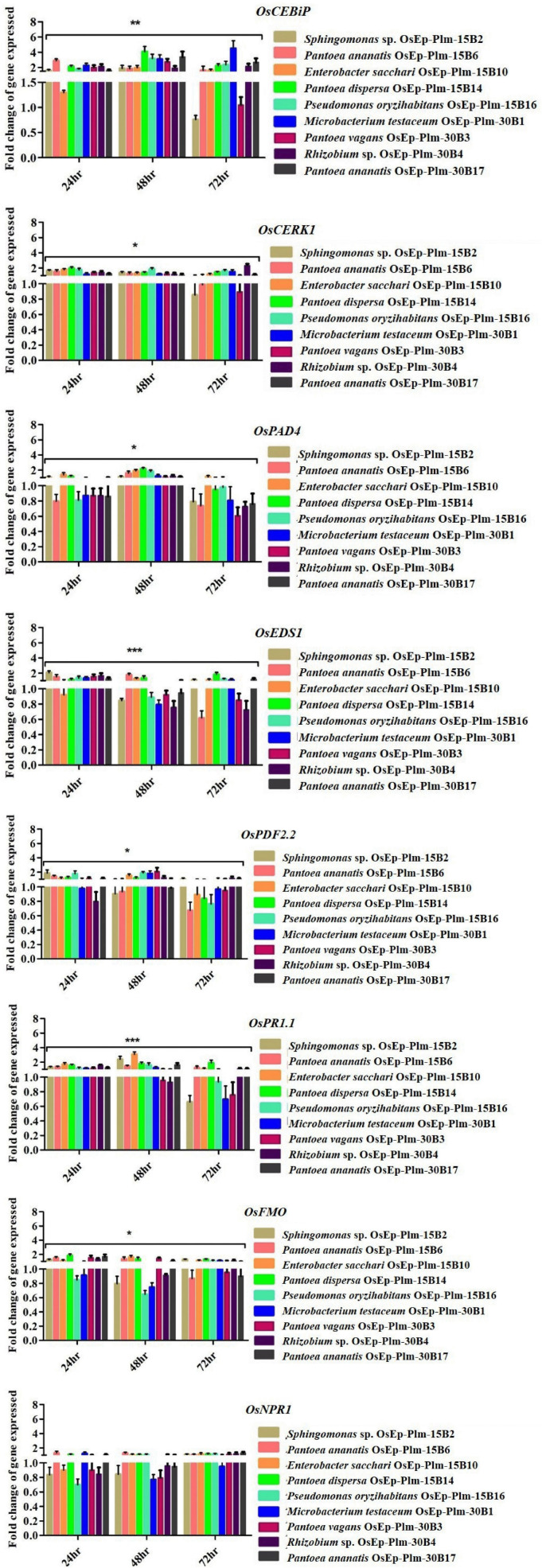
qPCR-based transcriptional analysis of defense gene expression in rice seedlings upon phyllobacterization. The fold change values obtained for the defense genes were imported into the GraphPad Prism program (https://www.graphpad.com/scientific-software/prism), and two-way ANOVA was conducted using Bonferroni *post-hoc* test for determining the statistical significance at *p ≤ 0.05, **p = 0.001, and ***p = 0.0001. Refer to Supplementary Table 10 for data pertaining to fold changes of gene expression.

The rice seedlings displaying the altered growth pattern were subjected to transcriptional analysis by qPCR. Defense genes such as *OsCEBiP*, *OsCERK1*, *OsPAD4*, *OsNPR1*, *OsEDS1*, *OsPDF2.2*, *Os*FMO1, and *OsPR1.1* showed marginal to a high level of expression in phyllobacterized rice seedlings compared with the untreated control compared with *OsActin* used as a reference gene. Interestingly, *Sphingomonas* sp. OsEp-Plm-15B2, *P. ananatis* OsEp-Plm-15B6, *E. sacchari* OsEp-Plm-15B10, *P. dispersa* OsEp-Plm-15B14, *P. oryzihabitans* OsEp-Plm-15B16, *M. testaceum* OsEp-Plm-30B1, *P. vagans* OsEp-Plm-30B3 *Rhizobium* sp. OsEp-Plm-30B4, and *P. ananatis* OsEp-Plm-30B17 sustained the overexpression of *OsCEBiP* in rice seedlings in all three-time points. *P. ananatis* OsEp-Plm-15B6 and *P. dispersa* OsEp-Plm-15B14 showed significant upregulation in almost all the defense-related genes at least for one-time point. *E. sacchari* OsEp-Plm-15B10 showed sustained upregulation of all the genes for 48 h after bacterial treatment. The epiphytic bacteria-mediated activation of defense genes was more pronounced during the early time points peaking at 48 hpi with a sharp drop at 72 h of bacterial interaction (Figure 5 and Supplementary Table 10).

### Hypersensitive Reaction on Tobacco

Rice phyllospheric bacteria upon infiltration in tobacco leaf did not trigger any necrotic reactions even after 48 h. The expression of quick necrosis due to the hypersensitive reaction was observed in the pathogen, *R. solanacearum*, which infiltrated a leaf indicating that the rice phyllosphere-associated bacterial isolates are non-pathogenic on plants (Supplementary Figure 9).

## Discussion

The foliar plant niches termed phyllosphere is one of the habitats for a diverse microbiota where the dynamic plant–microbe interactions are believed to impact plant performance in the ecosystem. With assistance from the microbial genome and their metabolic capabilities, the plants are exposed to a plethora of unpredictable, yet competitive or cooperative, microbial interactions (Vorholt, 2012; Hardoim et al., 2015; Reinhold-Hurek et al., 2015; Brader et al., 2017; Lemanceau et al., 2017). In recent years, the impact of microbe–microbe interactions on the host–microbial pathogen interaction outcomes is gaining the attention of researchers. Studies have shown that the microbiome structure, assemblage, and compositions are directly influenced by both micro and macro abiotic and biotic factors (Jacobs et al., 2005). Most of the previous microbiome studies have focused primarily on metagenomic or amplicon sequence surveys, and a few attempts have been made to validate the microbial mNGS datasets using classical microbiological culturomic tools. Strikingly, we have compared the phyllomicrobiome of blast disease-resistant and -susceptible rice genotypes planted in a blast endemic location in India. We recorded marginally high bacterial diversity and species richness on PRR78 that could be attributed to the innate susceptibility of the genotypes owing to the absence of functional NBS-LRR type of receptors termed as R-genes. Besides, the susceptible leaf showing early necrotic symptoms might have paved way for the inevitable microbial succession on the phyllosphere.

In the present work, we not only microbiologically validated the bacterial community structure in the phyllomicrobiome but also functionally characterized them for harnessing their antifungal and defense-inducing potential against rice blast disease. The NGS metabarcoding-based microbiome profiling revealed the predominance of phylum Proteobacteria on the rice phyllosphere. The predominance of Proteobacteria consisting of bacterial communities belonging to *Enterobacteriaceae* and *Pseudomonadaceae* is reported on the phyllosphere by many workers (Knief et al., 2012; Ren et al., 2014; Roman-Reyna et al., 2019; Yasmin et al., 2020). At the lower taxonomic genus hierarchy, *Pantoea* followed by *Pseudomonas* and *Enterobacter* were overrepresented on both the rice genotypes. Our findings are in agreement with many other reports that highlighted the ubiquitous occurrence of *Pantoea* as the most abundant bacterial genera on the phyllosphere (Cottyn et al., 2009; Cother et al., 2010; Kim et al., 2020; Stone and Jackson, 2020; Dhankhar et al., 2021). The microbiological investigation culminated in the isolation of 37 distinct morphotypes on the rice phyllosphere; here, the 4-week-old seedling harbored more bacterial morphotypes compared with the 15-day-old seedlings implying that microbial biomass on plant niches expands with the age of the plants. The morphotypes could be identified as belonging to 31 distinct amplicon groups in BOX-AIR-PCR fingerprinting based on shared amplicon profiles of the isolates. The BOX PCR DNA fingerprinting is one of the widely used molecular tools in bacterial typing and biogeography studies of microbial isolates (Versalovic et al., 1994; Brusetti et al., 2008).

The 16S rRNA gene sequence database search confirmed the identity of cultured bacterial isolates as belonging to *Acinetobacter*, *Enterobacter*, *Pantoea*, *Pseudomonas*, and *Sphingomonas* on blast-resistant and -susceptible genotypes. *Pantoea*, *Enterobacter*, *Microbacterium*, and *Curtobacterium* are recently reported as a member of the core microbiome of the rice leaf endosphere (Kumar et al., 2021). The genus *Microbacterium* too is frequently observed on the rice phyllosphere and spermosphere by several researchers in the past (Kaku et al., 2000; Leveau and Lindow, 2001; Midha et al., 2016). Characteristically, most of the phyllosphere bacterial genera produced shades of pigmentation as observed in *Acinetobacter* (pale brown), *Curtobacterium* (dark yellow), *Enterobacter* (pale brown to yellow), *Microbacterium* (yellow), *Pantoea* (yellow), *Pseudomonas* (pale brown to yellow), and *Sphingomonas* (dark yellow) (Supplementary Figures 4a–k). Production of dark pigmentation is one of the adaptive traits of bacteria and other microbes that encounter harsh environmental abiotic stresses like solar radiation and light on the phyllosphere (Green, 1992; Jacobs et al., 2005). Pigmentation on phyllospheric bacteria is believed to protect them from harmful ultraviolet radiation (Sundin and Jacobs, 1999). The rice phyllosphere is considered as the preferred habitat for yellow-pigmented *Pantoea* and pink-pigmented methylotrophs, which can survive under nutritional and moisture stress as well as can withstand harmful γ-ray radiation (Green, 1992). The effect of solar radiation on the composition and activities of the phyllosphere microbial community is reported by Carvalho and Castillo (2018).

The bacterial isolates showed antifungal activity on *M. oryzae* by their secreted compounds and volatile organic compounds, while *Enterobacter*, *Microbacterium*, *Pantoea*, *Pseudomonas*, and *Rhizobium* showed volatile mediated antifungal activity, and the *Acinetobacter* and *Sphingomonas* displayed secretory metabolite-mediated antagonism. The antagonistic potential of these bacterial species has been exploited for combating crop diseases caused by several fungal pathogens. For instance, the antagonistic potential of *Acinetobacter baumannii* (Liu et al., 2007), *Enterobacter* sp. (Gong et al., 2019), *M. testaceum* (Mannaa et al., 2017), *P. ananatis* (Gasser et al., 2012), *P. dispersa* (Jiang et al., 2019), *P. vagans* (Stockwell et al., 2010), *P. oryzihabitans* (Vagelas and Gowen, 2012; Rariz et al., 2017; Horuz and Aysan, 2018), and *Sphingomonas* sp. (Innerebner et al., 2011; Wachowska et al., 2013) is reported. Among them, *P. vagans* strain C9-1, isolated from apple has been registered by Nufarms America Inc., Burr Ridge, IL as “BlightBan C9-1” for the biological control of fire blight of apple caused by *Erwinia amylovora*. Isolates belonging to *Sphingomonas* have also been reported to promote plant growth, confer tolerance against abiotic stresses, and offer protection against plant pathogens (Luo et al., 2020; Turner, 2020).

Currently, growing evidence for microbe-induced seedling growth alteration as a phenotypic marker of activated innate immunity is published (Wang et al., 2021). We observed that the phyllosphere bacterial species in a density-dependent manner impacted the seed germination with consequent seedling growth alterations upon seed inoculation. The results are in agreement with the studies of Damodaran et al. (2013) and Zhu et al. (2017) who observed varying effects of endophytic and rhizosphere bacteria on seed germination. The seedling growth assay enabled us to identify the bacterial species that conferred immune competence in rice seedlings. Whereas the seedling growth was found inhibited at a higher dose (10^9^ cells per ml), the lower doses (10^6–8^ cells per ml) showed characteristic non-lethal seedling inhibition presumably owing to a tradeoff between growth and immunity. To confirm this, we performed the qPCR-based temporal transcriptional analysis of defense genes involved in innate immunity on phyllobacterized rice seedlings.

Phyllobacterized rice seedlings showed an elevated expression of defense genes, such as *OsCEBiP*, *OsCERK*, *OsPR1.1*, *OsNPR1*, *OsPDF2.2*, *OsFMO*, and *OsPAD4.* Significant up-regulation of almost all tested defense-related genes at least for a one-time point was shown by *P. ananatis* (OsEp-Plm-15B6) and *P. dispersa* (OsEp-Plm-15B14). Notably, *E. sacchari* (OsEp-Plm-15B10) showed sustained expression of all the genes 48 hpi. Among the genes, significant expression of *OsCEBiP* and *OsCERK* was observed in phyllobacterized rice seedlings. Both *OsCEBiP* and *OsCERK1* are reported to be activating MAMP-triggered immune (MTI) responses in plants upon chitin and peptidoglycan perception (Akamatsu et al., 2013; Kouzai et al., 2014). Defense genes, such as *OsPAD4* and *OsEDS1* participating in the jasmonic acid-mediated ISR, were also found induced in rice seedlings upon bacterization. Induction of *OsPAD4* contributes to the accumulation of rice phytoalexin, mamilactone-A, and contributes to basal resistance (Hasegawa et al., 2010; Ke et al., 2014, 2019). Marginal induction of *OsNPR1*, *OsFMO*, *OsPDF2.2*, and *OsPR1.1* was observed in phyllobacterized rice seedlings. Among them, *OsNPR1*—the key regulator of salicylic acid (SA)-mediated defense signaling is believed to control resource and energy redistribution during the defense reaction (Sugano et al., 2010). Likewise, *OsFMO1* is known to modulate systemic acquired resistance in plants against pathogens (Koch et al., 2006; Mishina and Zeier, 2006). While *OsPDF2.2* codes for antifungal plant defensin (Thomma et al., 2002), the *OsPR1.1* codes for an acidic pathogenesis-related protein to modulate SA-mediated systemic acquired resistance (Brader et al., 2017).

Phyllosphere bacterial species evaluated against rice blast under artificial epiphytotic trial in greenhouse showed a reduction in blast disease (50 % over mock) at all tested bacterial titers. Upon prophylactic foliar application (or phyllobacterization), the species belonging to *Pantoea*, *Enterobacter*, *Microbacterium*, *Pseudomonas*, *Sphingomonas*, and *Rhizobium* showed significant blast suppression at all tested doses. However, we could not observe any dosage response for enhanced blast suppression revealing the sufficiency of bacterial augmentation at 10^6–7^ cells per ml for reducing blast disease. Suppression of blast disease by *Bacillus*, *Streptomyces*, *Pseudomonas*, *Pantoea*, *Paenibacillus*, *Burkholderia*, *Enterobacter*, *Paraburkholderia*, and *Actinomycetes* was earlier reported in the literature (Gómez Expósito et al., 2017; Harsonowati et al., 2017; Schlatter et al., 2017). Rice blast suppression by *Microbacterium*, *Pseudomonas*, and *Stenotrophomonas* are attributed to the direct antifungal antibiosis and the indirect defense activation as evident from the expression of rice defense genes (Ashajyothi et al., 2020; Sahu et al., 2020). Recently, bacterial volatile belonging to pyrazines is reported to modulate defense against blast disease (Patel et al., 2020). Taken together, it is concluded that the enrichment of phyllosphere bacterial communities on the leaf can inflict antifungal antibiosis on *M. oryzae* and defense elicitation in rice to reduce the incidence and severity of blast disease.

Having confirmed the blast-suppressive potential of phyllosphere bacterial communities, we conducted a tobacco infiltration HR assay to ascertain the biosafety of the phyllosphere bacterial isolate (Klement, 1963). The tobacco HR assay has been recognized to test the pathogenic nature of plant-associated bacterial species (Kucheryava et al., 1999; Medina-Salazar et al., 2020; Sadeghi and Khodakaramian, 2020). None of the phyllosphere bacterial isolates showed any necrotic lesions, while comparing with *R. solanacearum* served as a positive check. Shades of faded yellowing observed with few phyllosphere bacterial isolates are indicative of activated defense.

**FIGURE 6:**
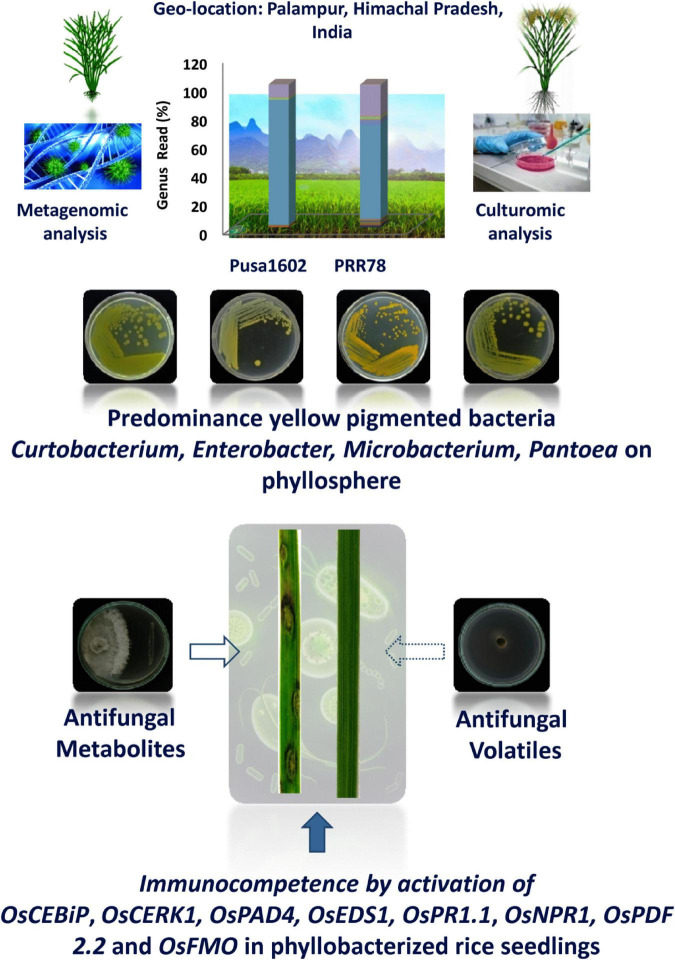
Phyllosphere microbiome-assisted suppression of rice blast disease. Blast suppressiveness by the predominant and pigmented bacterial isolates of phyllosphere can be attributed to both antifungal activity on *Magnaporthe oryzae* as well as induced defense in rice as evident from enhanced expression of many defense genes.

In conclusion, the phyllosphere bacterial communities suppressed the blast disease by the dual action of antifungal secreted and volatile metabolites as well as by microbe conferred immunocompetence (Figure 6). The present investigation on phyllosphere microbiome analysis of rice culminated in several potential hitherto unexplored bacterial communities for microbiome-assisted crop protection, especially against rice blast disease.

## Data Availability Statement

The datasets presented in this study can be found in online repositories. The names of the repository/repositories and accession number(s) can be found in the article/Supplementary Material.

## Author Contributions

KS conceptualized the study, developed the methodology, investigated and validated the study, performed the formal analysis, and wrote the original draft. AK conceptualized and investigated the study, provided the resources, wrote, reviewed, and edited the manuscript, and was in charge of the visualization, supervision, and project administration. AP, MK, and NS developed the methodology and validated the study. SM and BR performed the formal analysis. PE and NP validated the study. All authors read and approved the final manuscript.

## Conflict of Interest

The authors declare that the research was conducted in the absence of any commercial or financial relationships that could be construed as a potential conflict of interest. The reviewer KV declared a shared affiliation with the authors, to the handling editor at the time of the review.

## Publisher’s Note

All claims expressed in this article are solely those of the authors and do not necessarily represent those of their affiliated organizations, or those of the publisher, the editors and the reviewers. Any product that may be evaluated in this article, or claim that may be made by its manufacturer, is not guaranteed or endorsed by the publisher.
