# Supplementary material for: Integrated Metabarcoding and Culturomic-Based Microbiome Profiling of Rice Phyllosphere Reveal Diverse and Functional Bacterial Communities for Blast Disease Suppression
Source: Front Microbiol. 2021 Nov 30;12:780458. doi: 10.3389/fmicb.2021.780458 (PMC8669949; doi:10.3389/fmicb.2021.780458)
Supplement: Supplementary file 1 [file Data_Sheet_1.docx]

|  |  |
| --- | --- |
|  |  |

**Supplementary Fig. 1.** Relative abundance of phyllosphere bacterial communities at various taxonomic hierarchies on rice genotypes

| **Marker** | **15B1** | **15B2** | **15B3** | **15B4** | **15B5** | **15B6** | **15B7** | **15B8** | **15B9** | **15B10** | **15B11** | **15B12** | **15B13** | **15B14** | **15B15** | **15B16** | **30B1** | **30B2** | **30B3** | **30B4** | **30B5** | **30B6** | **30B7** | **30B8** | **30B9** | **30B10** | **30B11** | **30B12** | **30B13** | **30B14** | **30B15** | **30B16** | **30B17** | **30B18** | **30B19** | **30B20** | **30B21** | **Marker** |
| --- | --- | --- | --- | --- | --- | --- | --- | --- | --- | --- | --- | --- | --- | --- | --- | --- | --- | --- | --- | --- | --- | --- | --- | --- | --- | --- | --- | --- | --- | --- | --- | --- | --- | --- | --- | --- | --- | --- |

**Supplementary Fig. 2.** BOX PCR finger printing of cultured bacterial isolates of rice phyllosphere; M: DNA size marker; Lanes: Isolates of bacteria isolated from phyllosphere of rice leaf;

**Note:** Isolates **30B10, 30B11, 30B12, 30B13 and 30B16** shared all amplicons; one of them, 30B10, was considered as representative isolate for further work; **Note:** Isolates **30B14, 30B18 and 30B21** were found sharing all amplicons; one of them was considered as representative isolate for further work

| **Marker** | **15B1** | **15B2** | **15B3** | **15B4** | **15B5** | **15B6** | **15B7** | **15B8** | **15B9** | **15B10** | **15B11** | **15B12** | **15B13** | **15B14** | **15B15** | **15B16** | **30B1** | **30B2** | **30B3** | **30B4** | **30B5** | **30B6** | **30B7** | **30B8** | **30B9** | **30B10** | **30B14** | **30B15** | **30B17** | **30B19** | **30B20** | **Marker** |
| --- | --- | --- | --- | --- | --- | --- | --- | --- | --- | --- | --- | --- | --- | --- | --- | --- | --- | --- | --- | --- | --- | --- | --- | --- | --- | --- | --- | --- | --- | --- | --- | --- |

**Supplementary Fig. 3.** Amplification of 16S rDNA of bacterial isolates of rice phyllosphere

M: DNA size marker; Lane 1-31: Phyllosphere bacteria isolated from phyllosphere of rice leaf

| **Colonies of Rice Epiphytic*Acidovorax*** | | **Close up view of *Acidovorax* colonies** | | **Species identity**  **& Isolate name** |
| --- | --- | --- | --- | --- |
| **Nutrient Agar** | **Nutrient agar +**  **2, 3, 5 triphenyltetrazolium chloride** | **Nutrient Agar** | **Nutrient agar +**  **2, 3, 5 triphenyltetrazolium chloride** |  |
| **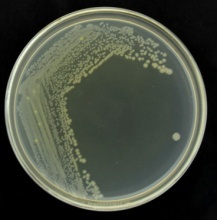** | **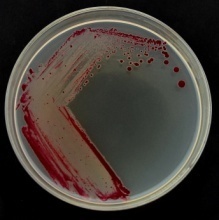** | **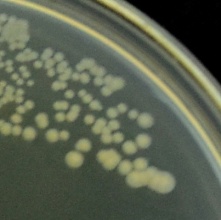** | **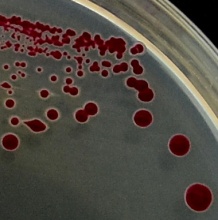** | ***Acidovorax avenae***  **(OsEp_Plm_15B4)** |

**Supplementary Fig. 4a. Colonies of cultured *Acidovorax*  species from rice phyllomicrobiome**

| **Colonies of Rice Epiphytic*Acinetobacter*** | | **Close up view of *Acinetobacter*  colonies** | | **Species identity**  **& Isolate name** |
| --- | --- | --- | --- | --- |
| **Nutrient Agar** | **Nutrient agar +**  **2, 3, 5 triphenyltetrazolium chloride** | **Nutrient Agar** | **Nutrient agar +**  **2, 3, 5 triphenyltetrazolium chloride** |  |
| **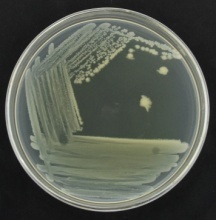** | **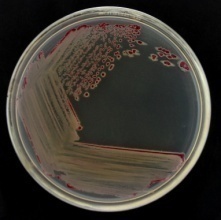** | **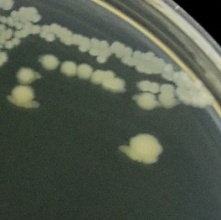** | **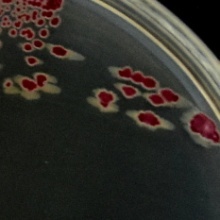** | ***Acinetobacter calcoaceticus***  **(OsEp_Plm_15B9)** |
| **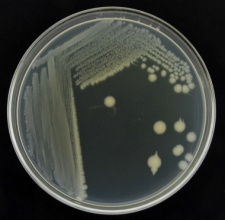** | **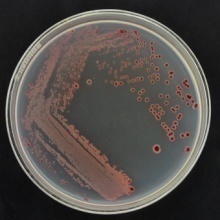** | **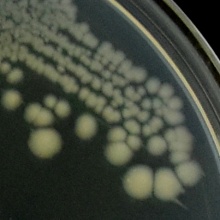** | **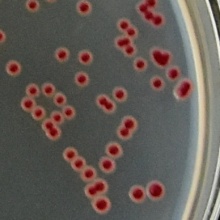** | ***Acinetobacter radioresistens***  **(OsEp_Plm_15B15)** |

**Supplementary Fig. 4b.Colonies of cultured *Acinetobacter*  species from rice phyllomicrobiome**

| **Colonies of Rice Epiphytic*Agrobacterium*** | | **Close up view of *Agrobacterium* colonies** | | **Species identity**  **& Isolate name** |
| --- | --- | --- | --- | --- |
| **Nutrient Agar** | **Nutrient agar +**  **2, 3, 5 triphenyl tetrazolium chloride** | **Nutrient Agar** | **Nutrient agar +**  **2, 3, 5 triphenyl tetrazolium chloride** |  |
| **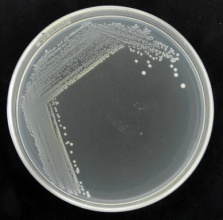** | **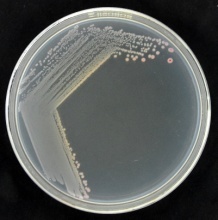** | **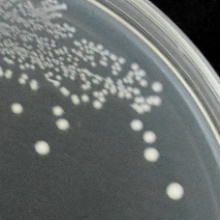** | **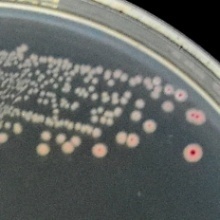** | ***Agrobacterium vitis* (OsEp_Plm_30B7)** |

**Supplementary Fig. 4c. Colonies of cultured *Agrobacterium* species from rice phyllomicrobiome**

| **Colonies of Rice Epiphytic*Curtobacterium*** | | **Close up view of *Curtobacterium*  colonies** | | **Species identity**  **& Isolate name** |
| --- | --- | --- | --- | --- |
| **Nutrient Agar** | **Nutrient agar +**  **2, 3, 5 triphenyl tetrazolium chloride** | **Nutrient Agar** | **Nutrient agar +**  **2, 3, 5 triphenyl tetrazolium chloride** |  |
| **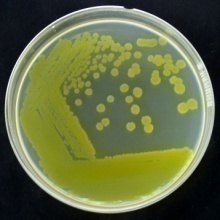** | **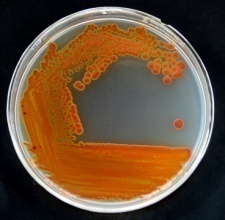** | **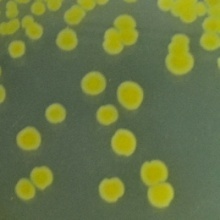** | **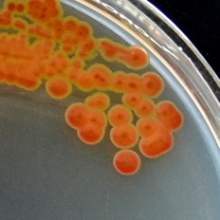** | ***Curtobacterium luteum* (OsEp_Plm_15B12)** |
| **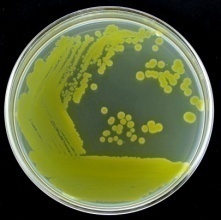** | **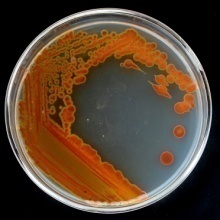** | **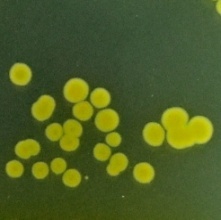** | **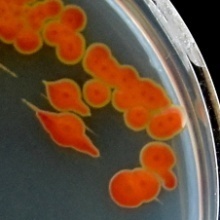** | ***Curtobacterium luteum* (OsEp_Plm_15B3)** |
| **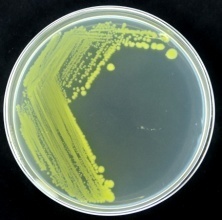** | **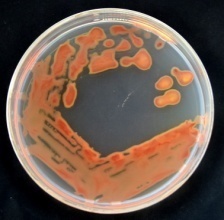** | **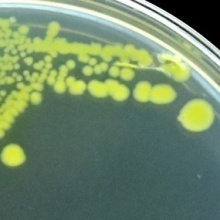** | **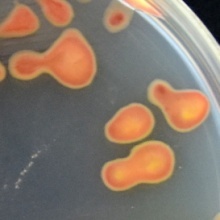** | ***Curtobacterium* sp. (OsEp_Plm_15B13)** |

**Supplementary Fig. 4d.Colonies of cultured *Curtobacterium* species from rice phyllomicrobiome**

| **Colonies of Rice Epiphytic*Enterobacter*** | | **Close up view of *Enterobacter*  colonies** | | **Species identity**  **& Isolate name** |
| --- | --- | --- | --- | --- |
| **Nutrient Agar** | **Nutrient agar +**  **2, 3, 5 triphenyl tetrazolium chloride** | **Nutrient Agar** | **Nutrient agar +**  **2, 3, 5 triphenyl tetrazolium chloride** |  |
| **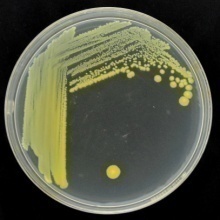** | **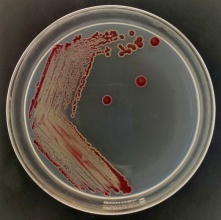** | **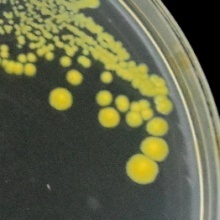** | **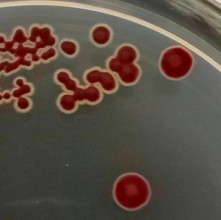** | ***Enterobacter cloacae*  (OsEp_Plm_30B10)** |
| **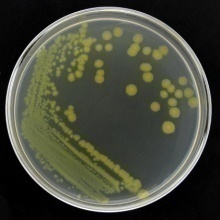** | **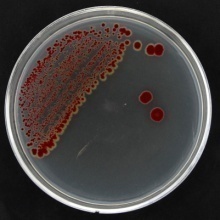** | **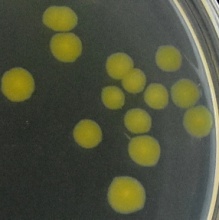** | **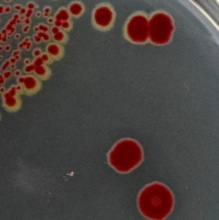** | ***Enterobacter ludwigii* (OsEp_Plm_30B20)** |
| **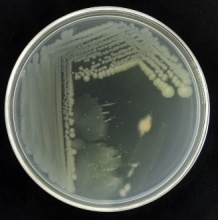** | **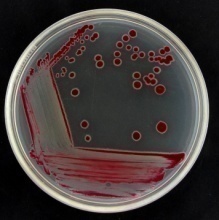** | **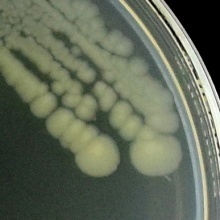** | **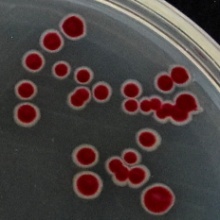** | ***Enterobacter sacchari* (OsEp_Plm_15B10)** |
| **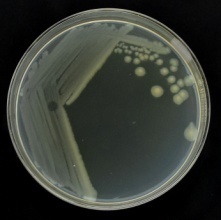** | **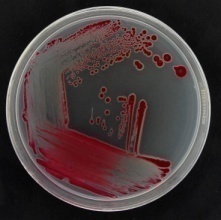** | **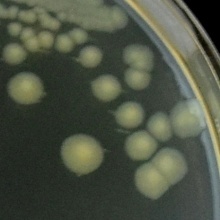** | **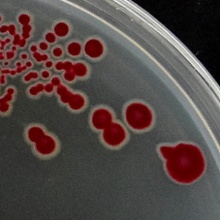** | ***Enterobacter* sp. (OsEp_Plm_15B11)** |

**Supplementary Fig. 4e.Colonies of cultured *Enterobacter* species from rice phyllomicrobiome**

| **Colonies of Rice Epiphytic *Enterococcus*** | | **Close up view of *Enterococcus*colonies** | | **Species identity**  **& Isolate name** |
| --- | --- | --- | --- | --- |
| **Nutrient Agar** | **Nutrient agar +**  **2, 3, 5 triphenyl tetrazolium chloride** | **Nutrient Agar** | **Nutrient agar +**  **2, 3, 5 triphenyl tetrazolium chloride** |  |
| **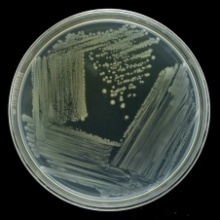** | **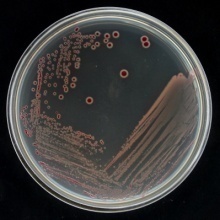** | **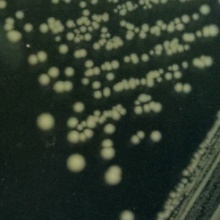** | **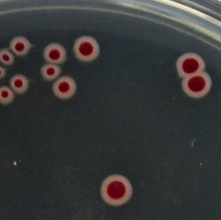** | ***Enterococcus faecium* (OsEp_Plm_15B7)** |

**Supplementary Fig. 4f.Colonies of cultured *Enterococcus* species from rice phyllomicrobiome**

| **Colonies of Rice Epiphytic*Microbacterium*** | | **Close up view of *Microbacterium*  colonies** | | **Species identity**  **& Isolate name** |
| --- | --- | --- | --- | --- |
| **Nutrient Agar** | **Nutrient agar +**  **2, 3, 5 triphenyl tetrazolium chloride** | **Nutrient Agar** | **Nutrient agar +**  **2, 3, 5 triphenyl tetrazolium chloride** |  |
| **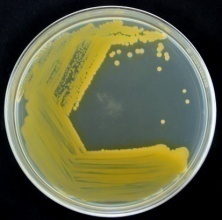** | **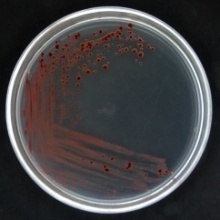** | **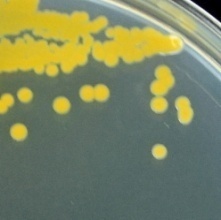** | **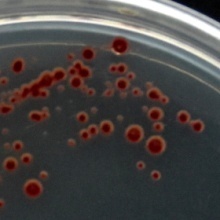** | ***Microbacterium sp.*  (OsEp_Plm_15B5)** |
| **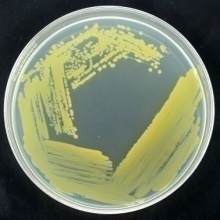** | **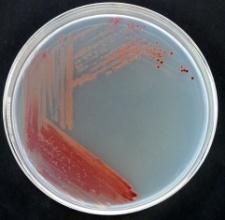** | **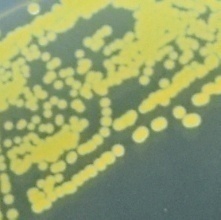** | **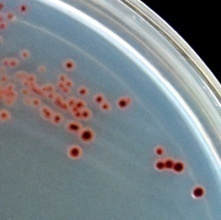** | ***Microbacterium testaceum* (OsEp_Plm_15B1)** |
| **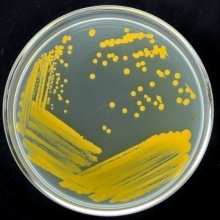** | **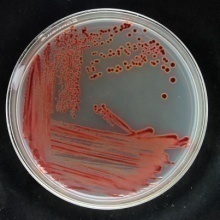** | **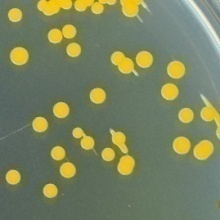** | **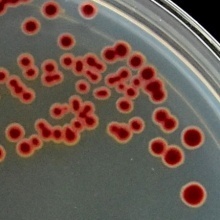** | ***Microbacterium testaceum* (OsEp_Plm_30B1)** |
| **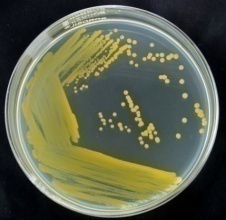** | **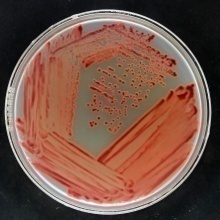** | **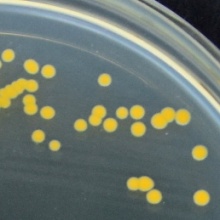** | **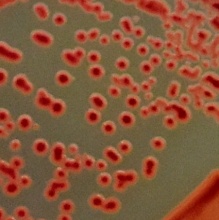** | ***Microbacterium testaceum* (OsEp_Plm_30B5)** |

**Supplementary Fig. 4g.Colonies of cultured *Microbacterium* species from rice phyllomicrobiome**

| **Colonies of Rice Epiphytic*Pantoea*** | | **Close up view of *Pantoea*  colonies** | | **Species identity**  **& Isolate name** |
| --- | --- | --- | --- | --- |
| **Nutrient Agar** | **Nutrient agar +**  **2, 3, 5 triphenyl tetrazolium chloride** | **Nutrient Agar** | **Nutrient agar +**  **2, 3, 5 triphenyl tetrazolium chloride** |  |
| **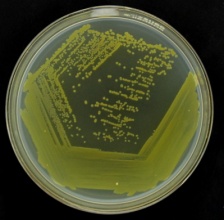** | **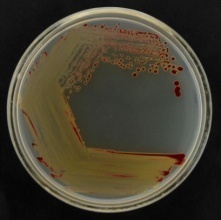** | **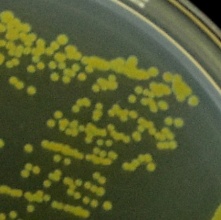** | **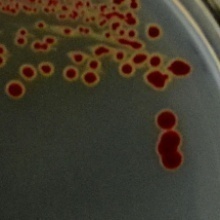** | ***Pantoea ananatis* (OsEp_Plm_15B6)** |
| **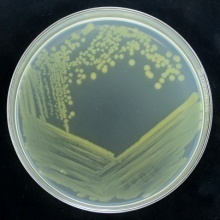** | **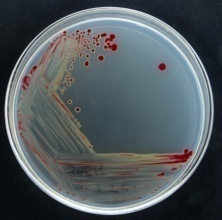** | **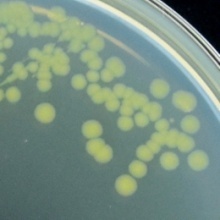** | **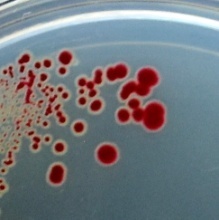** | ***Pantoea ananatis* (OsEp_Plm_30B15)** |
| **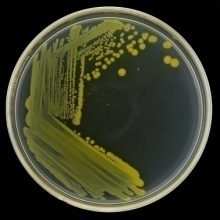** | **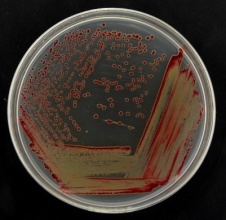** | **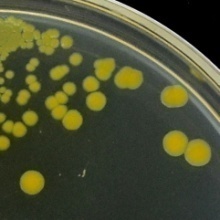** | **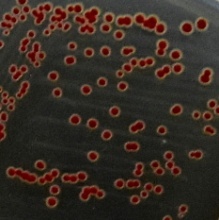** | ***Pantoea ananatis* (OsEp_Plm_30B17)** |
| **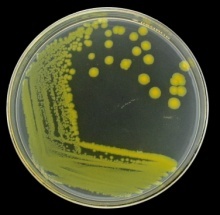** | **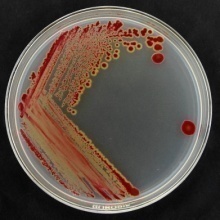** | **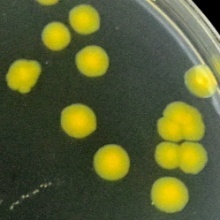** | **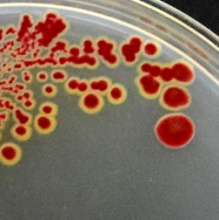** | ***Pantoea ananatis* (OsEp_Plm_30B19)** |
| **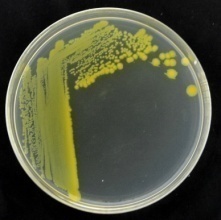** | **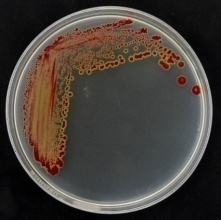** | **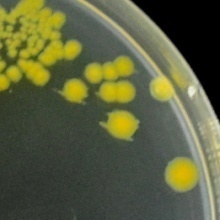** | **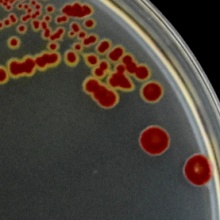** | ***Pantoea ananatis* (OsEp_Plm_30B2)** |
| **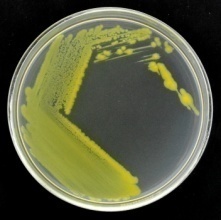** | **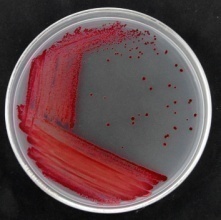** | **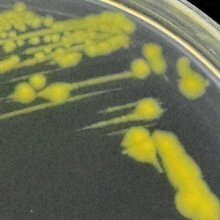** | **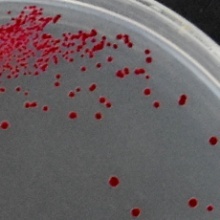** | ***Pantoea ananatis* (OsEp_Plm_30B6)** |
| **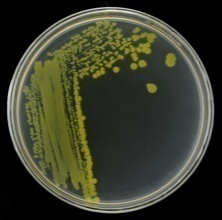** | **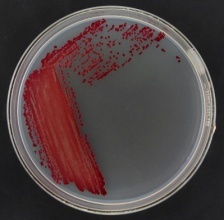** | **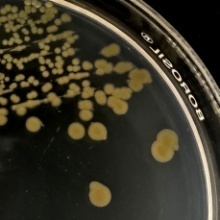** | **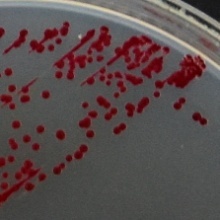** | ***Pantoea ananatis* (OsEp_Plm_30B8)** |
| **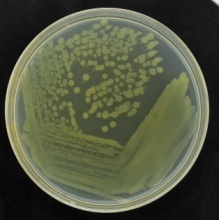** | **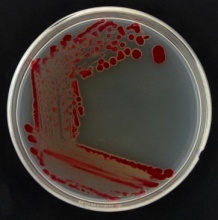** | **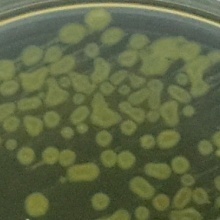** | **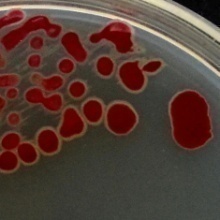** | ***Pantoea dispersa* (OsEp_Plm_15B14)** |
| **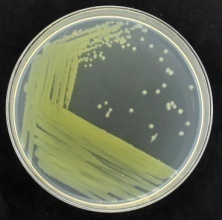** | **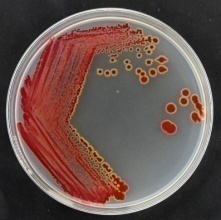** | **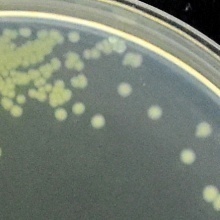** | **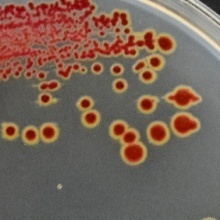** | ***Pantoea vagans* (OsEp_Plm_30B3)** |

**Supplementary Fig. 4h.Colonies of cultured *Pantoea* species from rice phyllomicrobiome**

| **Colonies of Rice Epiphytic *Pseudomonas*** | | **Close up view of *Pseudomonas* colonies** | | **Species identity**  **& Isolate name** |
| --- | --- | --- | --- | --- |
| **Nutrient Agar** | **Nutrient agar +**  **2, 3, 5 triphenyl tetrazolium chloride** | **Nutrient Agar** | **Nutrient agar +**  **2, 3, 5 triphenyl tetrazolium chloride** |  |
|  |  |  |  | ***Pseudomonas oryzihabitans* (OsEp_Plm_15B16)** |
|  |  |  |  | ***Pseudomonas sp.*  (OsEp_Plm_30B14)** |

**Supplementary Fig. 4i.Colonies of cultured *Pseudomonas* species from rice phyllomicrobiome**

| **Colonies of Rice Epiphytic *Rhizobium*** | | **Close up view of *Rhizobium* colonies** | | **Species identity**  **& Isolate name** |
| --- | --- | --- | --- | --- |
| **Nutrient Agar** | **Nutrient agar +**  **2, 3, 5 triphenyl tetrazolium chloride** | **Nutrient Agar** | **Nutrient agar +**  **2, 3, 5 triphenyl tetrazolium chloride** |  |
|  |  |  |  | ***Rhizobium sp.*  (OsEp_Plm_30B4)** |
|  |  |  |  | ***Rhizobium taibaishanense* (OsEp_Plm_15B8)** |

**Supplementary Fig. 4j.Colonies of cultured *Rhizobium* species from rice phyllomicrobiome**

| **Colonies of Rice Epiphytic *Sphingomonas*** | | **Close up view of *Sphingomonas*  colonies** | | **Species identity**  **& Isolate name** |
| --- | --- | --- | --- | --- |
| **Nutrient Agar** | **Nutrient agar +**  **2, 3, 5 triphenyl tetrazolium chloride** | **Nutrient Agar** | **Nutrient agar +**  **2, 3, 5 triphenyl tetrazolium chloride** |  |
|  |  |  |  | ***Sphingomonas pseudosanguinis* (OsEp_Plm_30B9)** |
|  |  |  |  | ***Sphingomonas* sp. (OsEp_Plm_15B2)** |

**Supplementary Fig. 4k.Colonies of cultured *Sphingomonas* species from rice phyllomicrobiome**

| ***Acidovorax*** | ***Acidovorax avenae* (OsEp_Plm_15B4)** |  |  |
| --- | --- | --- | --- |
|  |  |  |  |
| ***Acinetobacter*** | ***Acinetobacter calcoaceticus* (OsEp_Plm_15B9)** | ***Acinetobacter radioresistens* (OsEp_Plm_15B15)** |  |
|  |  |  |  |
| ***Agrobacterium*** | ***Agrobacterium vitis* (OsEp_Plm_30B7)** |  |  |
|  |  |  |  |
| ***Curtobacterium*** | ***Curtobacterium luteum* (OsEp_Plm_15B12)** | ***Curtobacterium luteum* (OsEp_Plm_15B3)** | ***Curtobacterium* sp. (OsEp_Plm_15B13)** |
|  |  |  |  |
| ***Enterobacter*** | ***Enterobacter cloacae*  (OsEp_Plm_30B10)** | ***Enterobacter ludwigii* (OsEp_Plm_30B20)** | ***Enterobacter sacchari* (OsEp_Plm_15B10)** |
|  |  |  |  |
|  | ***Enterobacter* sp. (OsEp_Plm_15B11)** |  |  |
|  |  |  |  |
| ***Enterococcus*** | ***Enterococcus faecium*(OsEp_Plm_15B7)** |  |  |
|  |  |  |  |
| ***Microbacterium*** | ***Microbacterium* sp. (OsEp_Plm_15B5)** | ***Microbacterium testaceum* (OsEp_Plm_15B1)** | ***Microbacterium testaceum* (OsEp_Plm_30B1)** |
|  |  |  |  |
|  | ***Microbacterium testaceum* (OsEp_Plm_30B5)** |  |  |
|  |  |  |  |
| ***Pantoea*** | ***Pantoea ananatis* (OsEp_Plm_15B6)** | ***Pantoea ananatis* (OsEp_Plm_30B15)** | ***Pantoea ananatis* (OsEp_Plm_30B17)** |
|  |  |  |  |
|  | ***Pantoea ananatis* (OsEp_Plm_30B19)** | ***Pantoea ananatis* (OsEp_Plm_30B2)** | ***Pantoea ananatis* (OsEp_Plm_30B6)** |
|  |  |  |  |
|  | ***Pantoea ananatis* (OsEp_Plm_30B8)** | ***Pantoea dispersa* (OsEp_Plm_15B14)** | ***Pantoea vagans* (OsEp_Plm_30B3)** |
|  |  |  |  |
| ***Pseudomonas*** | ***Pseudomonas oryzihabitans* (OsEp_Plm_15B16)** | ***Pseudomonas* sp. (OsEp_Plm_30B14)** |  |
|  |  |  |  |
| ***Rhizobium*** | ***Rhizobium* sp.**  **(OsEp_Plm_30B4)** | ***Rhizobium taibaishanense* (OsEp_Plm_15B8)** |  |
|  |  |  |  |
| ***Sphingomonas*** | ***Sphingomonas pseudosanguinis* (OsEp_Plm_30B9)** | ***Sphingomonas* sp*.*  (OsEp_Plm_15B2)** |  |
|  |  |  |  |
| **Control** | **Control** |  |  |
|  |  |  |  |

**Supplementary Fig. 5**. Secreted metabolite mediated *in vitro* antifungal activity of rice phyllosphere bacterial isolates on *Magnaporthe oryzae*

| ***Acidovorax*** | ***Acidovorax avenae* (OsEp_Plm_15B4)** |  |  |
| --- | --- | --- | --- |
|  |  |  |  |
| ***Acinetobacter*** | ***Acinetobacter calcoaceticus* (OsEp_Plm_15B9)** | ***Acinetobacter radioresistens* (OsEp_Plm_15B15)** |  |
|  |  |  |  |
| ***Agrobacterium*** | ***Agrobacterium vitis* (OsEp_Plm_30B7)** |  |  |
|  |  |  |  |
| ***Curtobacterium*** | ***Curtobacterium luteum* (OsEp_Plm_15B12)** | ***Curtobacterium luteum* (OsEp_Plm_15B3)** | ***Curtobacterium* sp. (OsEp_Plm_15B13)** |
|  |  |  |  |
| ***Enterobacter*** | ***Enterobacter cloacae*  (OsEp_Plm_30B10)** | ***Enterobacter ludwigii* (OsEp_Plm_30B20)** | ***Enterobacter sacchari* (OsEp_Plm_15B10)** |
|  |  |  |  |
|  | ***Enterobacter* sp. (OsEp_Plm_15B11)** |  |  |
|  |  |  |  |
| ***Enterococcus*** | ***Enterococcus faecium* (OsEp_Plm_15B7)** |  |  |
|  |  |  |  |
| ***Microbacterium*** | ***Microbacterium* sp. (OsEp_Plm_15B5)** | ***Microbacterium testaceum* (OsEp_Plm_15B1)** | ***Microbacterium testaceum* (OsEp_Plm_30B1)** |
|  |  |  |  |
|  | ***Microbacterium testaceum* (OsEp_Plm_30B5)** |  |  |
|  |  |  |  |
| ***Pantoea*** | ***Pantoea ananatis* (OsEp_Plm_15B6)** | ***Pantoea ananatis* (OsEp_Plm_30B15)** | ***Pantoea ananatis* (OsEp_Plm_30B17)** |
|  |  |  |  |
|  | ***Pantoea ananatis* (OsEp_Plm_30B19)** | ***Pantoea ananatis* (OsEp_Plm_30B2)** | ***Pantoea ananatis* (OsEp_Plm_30B6)** |
|  |  |  |  |
|  | ***Pantoea ananatis*(OsEp_Plm_30B8)** | ***Pantoea dispersa*(OsEp_Plm_15B14)** | ***Pantoea vagans* (OsEp_Plm_30B3)** |
|  |  |  |  |
| ***Pseudomonas*** | ***Pseudomonas oryzihabitans* (OsEp_Plm_15B16)** | ***Pseudomonas* sp. (OsEp_Plm_30B14)** |  |
|  |  |  |  |
| ***Rhizobium*** | ***Rhizobium* sp. (OsEp_Plm_30B4)** | ***Rhizobium taibaishanense* (OsEp_Plm_15B8)** |  |
|  |  |  |  |
| ***Sphingomonas*** | ***Sphingomonas pseudosanguinis* (OsEp_Plm_30B9)** | ***Sphingomonas* sp. (OsEp_Plm_15B2)** |  |
|  |  |  |  |
| **Control** | **Control** |  |  |
|  |  |  |  |

**Supplementary Fig. 6**. Volatile mediated antifungal activity of rice phyllosphere bacterial isolates on *Magnaporthe oryzae*

|  |  |  |
| --- | --- | --- |
| ***Pantoea ananatis***  **OsEp-Plm-15B6** | ***Enterobacter sacchari***  **OsEp-Plm-15B10** | ***Pantoea dispersa***  **OsEp-Plm-15B14** |
|  |  |  |
| ***Pseudomonas oryzihabitans***  **OsEp-Plm-15B16** | ***Microbacterium testaceum***  **OsEp-Plm-30B1** | ***Pantoea ananatis***  **OsEp-Plm-30B2** |
|  |  |  |
| ***Pantoea vagans***  **OsEp-Plm-30B3** | ***Rhizobium* sp.**  **OsEp-Plm-30B4** | ***Pantoea ananatis***  **OsEp-Plm-30B6** |
|  |  |  |
| ***Pantoea ananatis***  **OsEp-Plm-30B8** | ***Pantoea ananatis***  **OsEp-Plm-30B17** | ***Pantoea ananatis***  **OsEp-Plm-30B19** |

**Supplementary Fig. 7. Analysis of nature of BVC mediated growth inhibition of *Magnaporthe oryzae;*** Note: In each image; Top left plate: Mock from the beginning; Right plate: Mock from the day when volatile exposure in removed; Bottom plate: Plate incubated after removal of bacterial volatile exposure

| **10^6-7^** | **10^7-8^** | **10^8-9^** | **10^9-10^** | Bacterial isolate |
| --- | --- | --- | --- | --- |
|  |  |  |  | *Enterobacter sacchari*  OsEp-Plm-15B10 |
|  |  |  |  | *Microbacterium testaceum*  OsEp-Plm-30B1 |
|  |  |  |  | *Pantoea ananatis*  OsEp-Plm-15B6 |
|  |  |  |  | *Pantoea ananatis*  OsEp-Plm-30B17 |
|  |  |  |  | *Pantoea dispersa*  OsEp-Plm-15B14 |
|  |  |  |  | *Pantoea vagans*  OsEp-Plm-30B3 |
|  |  |  |  | *Pseudomonas oryzihabitans*  OsEp-Plm-15B16 |
|  |  |  |  | *Rhizobium* sp.  OsEp-Plm-30B4 |
|  |  |  |  | *Sphingomonas* sp.  OsEp-Plm-15B2 |
|  |  |  |  | Mock |

**Supplementary Fig. 8**. Effects of bacterial inoculation at various bacterial densities on rice seed germination

**Supplementary Fig. 9. Assay for hypersensitive reaction of rice phyllosphere associated bacteria on *Nicotiana tabaccum* (Arrow marks indicating site of infiltration)**

| **a.** | ***Enterobacter sacchari*OsEp-Plm-15B10** | **g.** | ***Pseudomonas oryzihabitans*OsEp-Plm-15B16** |
| --- | --- | --- | --- |
| **b.** | ***Microbacterium testaceum*OsEp-Plm-30B1** | **h.** | ***Rhizobium* sp. OsEp-Plm-30B4** |
| **c.** | ***Pantoea ananatis*OsEp-Plm-15B6** | **i.** | ***Sphingomonas* sp. OsEp-Plm-15B2** |
| **d.** | ***Pantoea ananatis*OsEp-Plm-30B17** | **j.** | **Negative control (Water infiltrated)** |
| **e.** | ***Pantoea dispersa*OsEp-Plm-15B14** | **k.** | **Positive control (*Ralstoniasolanacearum*)** |
| **f.** | ***Pantoea vagans*OsEp-Plm-30B3** |  |  |
