# Supplementary material for: Integrated Metabarcoding and Culturomic-Based Microbiome Profiling of Rice Phyllosphere Reveal Diverse and Functional Bacterial Communities for Blast Disease Suppression
Source: Front Microbiol. 2021 Nov 30;12:780458. doi: 10.3389/fmicb.2021.780458 (PMC8669949; doi:10.3389/fmicb.2021.780458)
Supplement: Supplementary file 2 [file Data_Sheet_2.docx]

**Supplementary Table 1.** Rice defense genes used for the qPCR analysis and their function

| **Gene** | **Characteristics and function** | **Reference** |
| --- | --- | --- |
| *OsCEBiP*  (Chitin Elicitor Binding Protein) | It is a Pattern Recognition Receptor (PRR) that detects the pathogen PAMP molecule chitin and activates the plant defense system. It is a plasma membrane protein that forms a receptor complex essential for fungal chitin-driven immune responses in rice. | Akamatsu et al. 2013 |
| *OsCERK1*  (Chitin Elicitor Receptor Kinase) | It is a rice receptor-like kinase (RLK) that mediates the signal of a fungal cell wall component chitin. It indispensable for chitin perception and participates in innate immunity. It can mediate the signaling pathways of both fungal and bacterial PAMP molecules. | Kouzai et al. 2014 |
| *OsPAD4* (Phytoalexin deficient 4) | Phytoalexin deficient 4 (PAD4) induces JA-dependent induced systemic resistance. It also plays an important role in the accumulation of JA and a terpenoid-type phytoalexin momilactone A (MOA). | Ke et al. 2014 |
| *OsEDS1* (Enhanced disease susceptibility 1) | Enhanced disease susceptibility 1 (EDS1) induces JA-dependent induced systemic resistance. | Ke et al. 2019 |
| *OsNPR1* (Non Repressor of Pathogenesis related Protein) | A central regulator of salicylic acid (SA)-mediated defense signaling  Reallocation of energy and resources during defense responses | Sugano et al. 2010 |
| *OsPDF2.2* (Plant Defensin-like protein 2) | Plant defensin inhibit the growth of a broad range of fungi | Thomma et al. 2002 |
| *OsFMO1* (Favin-dependent Monooxygenase 1) | An essential component for induced systemic acquired resistance (SAR) | Mishina et al 2006; Koch et al 2006 |
| *OsPR1.1* (Pathogenesis related protein 1) | Acidic pathogenesis-related protein 1, among the most abundant antimicrobial protein. It is also a marker for salicylic acid-mediated SAR | Breen et al 2017 |
| *Os*Actin | Housekeeping reference gene used in the analysis |  |

**Supplementary Table 2. List of the PCR primers used in the gene expression studies**

| **SN** | **Gene** |  | **Primer sequence(5→3)** | **Number of bases** | **Product size** |
| --- | --- | --- | --- | --- | --- |
| 1 | ***OsCEBiP* | Forward | GTGCGGAGAAGTCTGGAAAG | 20 | 131 |
|  |  | Reverse | TCCTGATTTCGCTTGCTTTT | 20 |  |
| 2 | ***OsCERK1* | Forward | AAGAACTACCGGGCAAAGGT | 20 | 244 |
|  |  | Reverse | GCCCCTTTGAATCACTTGAA | 20 |  |
| 3 | **OsPAD4* | Forward | TGCCGACTACCACCGAAAC | 19 | 61 |
|  |  | Reverse | CCGGCCATGGGTGATGTA | 18 |  |
| 4 | **OsEDS1* | Forward | TTGAATTTTGTCGTGCCAGTAGA | 23 | 63 |
|  |  | Reverse | GGCAGATGCAAGCGGAGTAA | 20 |  |
| 5 | **OsNPR1* | Forward | AAACAAAGGAGCAGCTGTATCACA | 24 | 66 |
|  |  | Reverse | CTCCGGCAGATACTCATTGCA | 21 |  |
| 6 | **OsPDF2.2* | Forward | CCACAGGTTCAAGGGCATGT | 20 | 63 |
|  |  | Reverse | CTCTCCGTCCTGCACACGTT | 20 |  |
| 7 | **OsFMO1* | Forward | CAGTGGAGTGCCCAACATACC | 21 | 65 |
|  |  | Reverse | CCTGGCCATCAAATGCTTCT | 20 |  |
| 8 | **Os PR1.1* | Forward | GGAGGCATCCAAGCTAGCAA | 20 | 80 |
|  |  | Reverse | GGGCATCGGAGCAGTGAA | 18 |  |
| 9 | **OsActin* | Forward | CAGCCACACTGTCCCCATCTA | 21 | 67 |
|  |  | Reverse | AGCAAGGTCGAGACGAAGGA | 20 |  |

*Patel 2018, Ashajyothi et al 2020

**Primers designed using online Platform Primer3Plus <http://www.bioinformatics.nl/cgi-bin/primer3plus/primer3plus.cgi>

**Supplementary Table 3.** Phyllomicrobiome profiles of blast resistant and susceptible rice genotypes grown in blast endemic location at Palampur, Himachal Pradesh, India

| *Bacterial Taxa | Blast Resistant Pusa1602 | | Blast Susceptible PRR78 | |
| --- | --- | --- | --- | --- |
|  | Read | Read % | Read | Read % |
| **Phylum** |  |  |  |  |
| Actinobacteria | 295 | 0.061 | 1004 | 0.485 |
| Bacteroidetes | 172 | 0.036 | 8721 | 4.211 |
| Deinococcus-Thermus | 31 | 0.006 | 28 | 0.014 |
| Euryarchaeota | 0 | 0.000 | 23 | 0.011 |
| Firmicutes | 507 | 0.105 | 5501 | 2.656 |
| Fusobacteria | 11 | 0.002 | 1420 | 0.686 |
| **Proteobacteria** | **440098** | **91.066** | **146595** | **70.792** |
| Spirochaetes | 0 | 0.000 | 83 | 0.040 |
| Tenericutes | 0 | 0.000 | 43 | 0.021 |
| Unclassified (derived from Bacteria) | 42158 | 8.723 | 43660 | 21.084 |
| **Class** |  |  |  |  |
| Actinobacteria | 295 | 0.061 | 1004 | 0.485 |
| Alphaproteobacteria | 59 | 0.012 | 31 | 0.015 |
| Bacilli | 116 | 0.024 | 3738 | 1.805 |
| Bacteroidia | 139 | 0.029 | 7797 | 3.766 |
| Betaproteobacteria | 100 | 0.021 | 502 | 0.242 |
| Clostridia | 383 | 0.079 | 1065 | 0.514 |
| Cytophagia | 19 | 0.004 | 0 | 0.000 |
| Deinococci | 31 | 0.006 | 28 | 0.014 |
| Epsilonproteobacteria | 0 | 0.000 | 101 | 0.049 |
| Flavobacteria | 0 | 0.000 | 885 | 0.427 |
| Fusobacteria | 11 | 0.002 | 1420 | 0.686 |
| **Gammaproteobacteria** | **439906** | **91.031** | **145961** | **70.491** |
| Halobacteria | 0 | 0.000 | 23 | 0.011 |
| Mollicutes | 0 | 0.000 | 43 | 0.021 |
| Negativicutes | 0 | 0.000 | 691 | 0.334 |
| Spirochaetes | 0 | 0.000 | 83 | 0.040 |
| Unclassified (derived from Bacteria) | 42158 | 8.724 | 43660 | 21.085 |
| Unclassified (derived from Bacteroidetes) | 0 | 0.000 | 32 | 0.015 |
| Unclassified (derived from Proteobacteria) | 32 | 0.007 | 0 | 0.000 |
| **Order** |  |  |  |  |
| Actinomycetales | 288 | 0.060 | 982 | 0.474 |
| Alteromonadales | 91 | 0.019 | 0 | 0.000 |
| Bacillales | 61 | 0.013 | 1230 | 0.594 |
| **Bacteroidales** | **139** | **0.029** | **7797** | **3.766** |
| Burkholderiales | 16 | 0.003 | 17 | 0.008 |
| Campylobacterales | 0 | 0.000 | 101 | 0.049 |
| Caulobacterales | 27 | 0.006 | 0 | 0.000 |
| Clostridiales | 383 | 0.079 | 1065 | 0.514 |
| Coriobacteriales | 0 | 0.000 | 22 | 0.011 |
| Cytophagales | 19 | 0.004 | 0 | 0.000 |
| Deinococcales | 31 | 0.006 | 28 | 0.014 |
| **Enterobacter iales** | **431232** | **89.243** | **140804** | **68.005** |
| Entomoplasmatales | 0 | 0.000 | 27 | 0.013 |
| Flavobacteriales | 0 | 0.000 | 885 | 0.427 |
| Fusobacteriales | 11 | 0.002 | 1420 | 0.686 |
| Halobacteriales | 0 | 0.000 | 23 | 0.011 |
| Lactobacillales | 55 | 0.011 | 2508 | 1.211 |
| Mycoplasmatales | 0 | 0.000 | 16 | 0.008 |
| Neisseriales | 0 | 0.000 | 461 | 0.223 |
| Pasteurellales | 0 | 0.000 | 521 | 0.252 |
| **Pseudomonadales** | **8455** | **1.750** | **4608** | **2.226** |
| Rhizobiales | 25 | 0.005 | 22 | 0.011 |
| Selenomonadales | 0 | 0.000 | 691 | 0.334 |
| Spirochaetales | 0 | 0.000 | 83 | 0.040 |
| Xanthomonadales | 105 | 0.022 | 23 | 0.011 |
| Unclassified (derived from Bacteria) | 42158 | 8.725 | 43660 | 21.087 |
| Unclassified (derived from Bacteroidetes) | 0 | 0.000 | 32 | 0.015 |
| Unclassified (derived from Betaproteobacteria) | 84 | 0.017 | 24 | 0.012 |
| Unclassified (derived from Proteobacteria) | 32 | 0.007 | 0 | 0.000 |
| Family |  |  |  |  |
| Actinomycetaceae | 0 | 0.000 | 32 | 0.015 |
| Aerococcaceae | 0 | 0.000 | 1523 | 0.736 |
| Alteromonadaceae | 91 | 0.019 | 0 | 0.000 |
| Bacillaceae | 51 | 0.011 | 10 | 0.005 |
| Bacteroidaceae | 116 | 0.024 | 346 | 0.167 |
| Burkholderiaceae | 0 | 0.000 | 11 | 0.005 |
| Campylobacteraceae | 0 | 0.000 | 101 | 0.049 |
| Carnobacteriaceae | 0 | 0.000 | 807 | 0.390 |
| Caulobacteraceae | 27 | 0.006 | 0 | 0.000 |
| Clostridiaceae | 144 | 0.030 | 206 | 0.100 |
| Clostridiales Family XI. IncertaeSedis | 0 | 0.000 | 131 | 0.063 |
| Coriobacteriaceae | 0 | 0.000 | 22 | 0.011 |
| Corynebacteriaceae | 0 | 0.000 | 32 | 0.015 |
| Cytophagaceae | 19 | 0.004 | 0 | 0.000 |
| Deinococcaceae | 31 | 0.006 | 28 | 0.014 |
| **Enterobacter iaceae** | **431232** | **89.257** | **140804** | **68.017** |
| Enterococcaceae | 40 | 0.008 | 143 | 0.069 |
| Entomoplasmataceae | 0 | 0.000 | 27 | 0.013 |
| Eubacteriaceae | 0 | 0.000 | 76 | 0.037 |
| Flavobacteriaceae | 0 | 0.000 | 885 | 0.428 |
| Fusobacteriaceae | 11 | 0.002 | 1420 | 0.686 |
| Geodermatophilaceae | 29 | 0.006 | 11 | 0.005 |
| Halobacteriaceae | 0 | 0.000 | 23 | 0.011 |
| Intrasporangiaceae | 17 | 0.004 | 0 | 0.000 |
| Kineosporiaceae | 35 | 0.007 | 139 | 0.067 |
| Lachnospiraceae | 90 | 0.019 | 372 | 0.180 |
| Lactobacillaceae | 0 | 0.000 | 21 | 0.010 |
| Methylobacteriaceae | 24 | 0.005 | 18 | 0.009 |
| Microbacteriaceae | 67 | 0.014 | 30 | 0.014 |
| Micrococcaceae | 34 | 0.007 | 700 | 0.338 |
| Moraxellaceae | 22 | 0.005 | 382 | 0.185 |
| Mycoplasmataceae | 0 | 0.000 | 16 | 0.008 |
| Neisseriaceae | 0 | 0.000 | 461 | 0.223 |
| Nocardiaceae | 36 | 0.007 | 11 | 0.005 |
| Nocardioidaceae | 38 | 0.008 | 10 | 0.005 |
| Oxalobacteraceae | 13 | 0.003 | 0 | 0.000 |
| Pasteurellaceae | 0 | 0.000 | 521 | 0.252 |
| Peptostreptococcaceae | 0 | 0.000 | 33 | 0.016 |
| Porphyromonadaceae | 16 | 0.003 | 6386 | 3.085 |
| Prevotellaceae | 0 | 0.000 | 1064 | 0.514 |
| **Pseudomonadaceae** | **8433** | **1.745** | **4226** | **2.041** |
| Spirochaetaceae | 0 | 0.000 | 83 | 0.040 |
| Streptococcaceae | 0 | 0.000 | 14 | 0.007 |
| Veillonellaceae | 0 | 0.000 | 691 | 0.334 |
| Xanthomonadaceae | 105 | 0.022 | 23 | 0.011 |
| Unclassified (derived from Bacillales) | 0 | 0.000 | 1213 | 0.586 |
| Unclassified (derived from Bacteria) | 42158 | 8.726 | 43660 | 21.091 |
| Unclassified (derived from Bacteroidetes) | 0 | 0.000 | 32 | 0.015 |
| Unclassified (derived from Betaproteobacteria) | 84 | 0.017 | 24 | 0.012 |
| Unclassified (derived from Clostridiales) | 142 | 0.029 | 244 | 0.118 |
| Unclassified (derived from Proteobacteria) | 32 | 0.007 | 0 | 0.000 |
| Genus |  |  |  |  |
| *Abiotrophia* | 0 | 0.000 | **1522** | 0.736 |
| *Acinetobacter* | 22 | 0.005 | 0 | 0.000 |
| *Actinobacillus* | 0 | 0.000 | 46 | 0.022 |
| *Actinomyces* | 0 | 0.000 | 32 | 0.015 |
| *Alishewanella* | 91 | 0.019 | 0 | 0.000 |
| *Atopobium* | 0 | 0.000 | 22 | 0.011 |
| *Bacillus* | 49 | 0.010 | 0 | 0.000 |
| *Bacteroides* | 116 | 0.024 | 346 | 0.167 |
| *Barnesiella* | 12 | 0.002 | 21 | 0.010 |
| *Blautia* | 21 | 0.004 | 44 | 0.021 |
| *Brevundimonas* | 27 | 0.006 | 0 | 0.000 |
| ***Buttiauxella*** | **1210** | **0.250** | **0** | **0.000** |
| *Butyricicoccus* | 0 | 0.000 | 34 | 0.016 |
| *Butyrivibrio* | 0 | 0.000 | 134 | 0.065 |
| *Campylobacter* | 0 | 0.000 | 101 | 0.049 |
| *Capnocytophaga* | 0 | 0.000 | 518 | 0.250 |
| *Clostridium* | 144 | 0.030 | 172 | 0.083 |
| *Corynebacterium* | 0 | 0.000 | 32 | 0.015 |
| *Cupriavidus* | 0 | 0.000 | 10 | 0.005 |
| *Curtobacterium* | 38 | 0.008 | 10 | 0.005 |
| *Deinococcus* | 31 | 0.006 | 28 | 0.014 |
| *Duganella* | 11 | 0.002 | 0 | 0.000 |
| *Elizabethkingia* | 0 | 0.000 | 206 | 0.100 |
| ***Enterobacter*** | **4071** | **0.843** | **1270** | **0.614** |
| *Enterococcus* | 40 | 0.008 | 142 | 0.069 |
| ***Erwinia*** | **501** | **0.104** | **264** | **0.128** |
| *Escherichia* | 0 | 0.000 | **755** | 0.365 |
| *Eubacterium* | 0 | 0.000 | 76 | 0.037 |
| *Flavobacterium* | 0 | 0.000 | 152 | 0.073 |
| *Fusobacterium* | 0 | 0.000 | **1093** | 0.528 |
| *Gemella* | 0 | 0.000 | **1213** | 0.586 |
| *Geodermatophilus* | 29 | 0.006 | 11 | 0.005 |
| *Granulicatella* | 0 | 0.000 | **807** | 0.390 |
| *Haemophilus* | 0 | 0.000 | 475 | 0.230 |
| *Hymenobacter* | 19 | 0.004 | 0 | 0.000 |
| *Janibacter* | 13 | 0.003 | 0 | 0.000 |
| *Kineococcus* | 35 | 0.007 | 139 | 0.067 |
| *Kingella* | 0 | 0.000 | 11 | 0.005 |
| *Klebsiella* | 402 | 0.083 | **769** | 0.372 |
| *Kluyvera* | 0 | 0.000 | 135 | 0.065 |
| *Lactobacillus* | 0 | 0.000 | 21 | 0.010 |
| *Leptotrichia* | 0 | 0.000 | 220 | 0.106 |
| *Megasphaera* | 0 | 0.000 | 29 | 0.014 |
| *Mesoplasma* | 0 | 0.000 | 27 | 0.013 |
| *Methylobacterium* | 24 | 0.005 | 18 | 0.009 |
| *Microbacterium* | 27 | 0.006 | 16 | 0.008 |
| *Micrococcus* | 25 | 0.005 | 0 | 0.000 |
| *Moraxella* | 0 | 0.000 | 382 | 0.185 |
| *Mycoplasma* | 0 | 0.000 | 16 | 0.008 |
| *Neisseria* | 0 | 0.000 | 450 | 0.217 |
| *Nocardioides* | 35 | 0.007 | 0 | 0.000 |
| *Oribacterium* | 0 | 0.000 | 21 | 0.010 |
| ***Pantoea*** | **423893** | **87.751** | **137297** | **66.357** |
| *Peptostreptococcus* | 0 | 0.000 | 33 | 0.016 |
| ***Porphyromonas*** | 0 | 0.000 | **6355** | **3.071** |
| *Prevotella* | 0 | 0.000 | **1064** | 0.514 |
| ***Pseudomonas*** | **8433** | **1.746** | **4226** | **2.042** |
| *Rahnella* | 0 | 0.000 | 12 | 0.006 |
| *Rhodococcus* | 36 | 0.007 | 11 | 0.005 |
| *Robinsoniella* | 0 | 0.000 | 76 | 0.037 |
| *Roseburia* | 10 | 0.002 | 10 | 0.005 |
| *Rothia* | 0 | 0.000 | **696** | 0.336 |
| *Salmonella* | 141 | 0.029 | 97 | 0.047 |
| *Selenomonas* | 0 | 0.000 | 46 | 0.022 |
| *Serratia* | 94 | 0.019 | 0 | 0.000 |
| *Stenotrophomonas* | 0 | 0.000 | 21 | 0.010 |
| *Streptobacillus* | 0 | 0.000 | 107 | 0.052 |
| *Streptococcus* | 0 | 0.000 | 14 | 0.007 |
| *Treponema* | 0 | 0.000 | 83 | 0.040 |
| *Veillonella* | 0 | 0.000 | **608** | 0.294 |
| *Xanthomonas* | 99 | 0.020 | 0 | 0.000 |
| *Yersinia* | 0 | 0.000 | 28 | 0.014 |
| Unclassified  (derived from Bacteria) | 42158 | 8.727 | 43660 | 21.101 |
| Unclassified  (derived from Bacteroidetes) | 0 | 0.000 | 32 | 0.015 |
| Unclassified  (derived from Betaproteobacteria) | 84 | 0.017 | 24 | 0.012 |
| Unclassified  (derived from Clostridiales Family XI. IncertaeSedis) | 0 | 0.000 | 131 | 0.063 |
| Unclassified  (derived from Clostridiales) | 121 | 0.025 | 200 | 0.097 |
| Unclassified  (derived from Enterobacteriaceae) | 907 | 0.188 | 175 | 0.085 |
| Unclassified  (derived from Lachnospiraceae) | 65 | 0.013 | 111 | 0.054 |
| Unclassified  (derived from Proteobacteria) | 32 | 0.007 | 0 | 0.000 |

*Metagenomic hits with more than 10 reads are only considered for analysis.

**Supplementary Table 4.** Morphotyping and population size of epiphytic bacteria on phyllosphere of 15 and 30 days old blast resistant and susceptible rice genotype

| **Isolate** | **Colony morphology** | **Population size of epiphytic bacteria (Log CFU g^_1^)** | | **Isolate** | **Colony morphology** | **Population size of epiphytic bacteria**  **(Log CFU g^_1^)** | | | |  |
| --- | --- | --- | --- | --- | --- | --- | --- | --- | --- | --- |
|  |  | **PRR78** | **Pusa 1602** |  |  | **PRR78** | **Pusa 1602** | | | |
| 15B1 | Punctiform, dark red | 5.56 | 5.03 | 30B1 | Punctiform, dark red | 5.60 | | 5.53 |  |  |
| 15B2 | Punctiform, light red with white hallow | 4.82 | 4.17 | 30B2 | Punctiform, light red with white hallow | 3.56 | | 3.48 |  |  |
| 15B3 | Punctiform, light red with yellow hallow | 4.43 | 2.14 | 30B3 | Punctiform, light red with yellow hallow | 4.80 | | 4.38 |  |  |
| 15B4 | Small circular, dark red, entire | 5.37 | 4.54 | 30B4 | Small circular, dark red, entire | 5.43 | | 5.13 |  |  |
| 15B5 | Small circular, opaque white with red center, entire | 4.56 | 3.39 | 30B5 | Small circular, opaque white with red center, entire | 3.17 | | 3.81 |  |  |
| 15B6 | Large, opaque white, irregular | 4.39 | 2.71 | 30B6 | Large, opaque white, irregular | 2.77 | | 4.33 |  |  |
| 15B7 | Moderate, red with opaque margin, entire | 2.16 | 3.65 | 30B7 | Moderate, red with opaque margin, entire | 2.47 | | 3.87 |  |  |
| 15B8 | Moderate, dark red, with metallic shine, entire | 2.81 | 0.00 | 30B8 | Moderate, dark red, with metallic shine, entire | 3.57 | | 0.00 |  |  |
| 15B9 | Small dark red with dark yellow border, entire | 3.17 | 0.00 | 30B9 | Small dark red with dark yellow border, entire | 1.82 | | 0.00 |  |  |
| 15B10 | Moderate, translucent, convex surface, irregular margin | 4.60 | 3.36 | 30B10 | Moderate, translucent, convex surface, irregular margin | 3.31 | | 3.74 |  |  |
| 15B11 | Large, translucent, lobate margin | 4.13 | 1.55 | 30B11 | Large, translucent, lobate margin | 3.39 | | 1.59 |  |  |
| 15B12 | Large, opaque white, filamentous margin | 2.50 | 1.55 | 30B12 | Large, opaque white, filamentous margin | 3.00 | | 0.00 |  |  |
| 15B13 | Moderate, pinkish white, filamentous border | 4.22 | 0.00 | 30B13 | Moderate, pinkish white, filamentous border | 4.50 | | 0.00 |  |  |
| 15B14 | Small greenish white with red centre, entire | 1.92 | 2.45 | 30B14 | Small greenish white with red centre, entire | 1.62 | | 2.10 |  |  |
| 15B15 | Moderate opaque yellow with red centre, metallic shine, entire | 3.61 | 3.49 | 30B15 | Moderate opaque yellow with red centre, metallic shine, entire | 4.44 | | 2.13 |  |  |
| 15B16 | Moderate translucent entire with metallic shine | 4.38 | 0.00 | 30B16 | Moderate translucent entire with metallic shine | 3.80 | | 0.00 |  |  |
| - | - | - | - | 30B17 | Large, light pink with dark red center, flat surface entire margin | 1.55 | | 3.08 |  |  |
| - | - | - | - | 30B18 | Moderate red with opaque margin, entire | 2.69 | | 2.61 |  |  |
| - | - | - | - | 30B19 | Small boat shape dark red with brown hallow | 4.40 | | 3.55 |  |  |
| - | - | - | - | 30B20 | Large, opaque white with rhizoidal margin | 1.95 | | 0.00 |  |  |
| - | - | - | - | 30B21 | Moderate, opaque purple with irregular margin | 3.71 | | 3.01 |  |  |
|  | Mean | 3.91 | 2.38 |  | Mean | 3.41 | | 2.50 |  |  |
|  |  | Cultivar | Bacteria |  |  | Cultivar | | Bacteria |  |  |
|  | F cal | 125.2 | 18.78 |  | F cal | 44.71 | | 16.61 |  |  |
|  | F tab | 3.88 | 1.71 |  | F tab | 3.87 | | 1.6 |  |  |

CFU/ml= (Number of colonies) X (Dilution factor)/Volume plated in mL

**Supplementary Table 5.** Diversity indices of phyllosphere bacterial species on rice seedling grown in Palampur, Himachal Pradesh, India

| **Rice phyllosphere** | **Parameters** | **15^th^ Day** | | **30^th^ Day** | |
| --- | --- | --- | --- | --- | --- |
|  |  | Resistant  Genotype  (Pusa 1602) | Susceptible genotype  (PRR78) | Resistant  Genotype  (Pusa 1602) | Susceptible genotype  (PRR78) |
| **Palampur 2014** | Shannon wiener diversity index | **1.72** | **1.82** | **1.48** | **1.63** |
|  | Species richness | **12.0** | **16.0** | **12.0** | **15.0** |

**Supplementary Table 6.** Analysis of nature of volatile mediated mycelial inhibition of *Magnaporthe oryzae*

| **Isolate** | **Bacterial Species** | **Inhibition by BVC (%)** | **Re-growth of *Magnaporthe oryzae* (%)** | **Nature of BVC** |
| --- | --- | --- | --- | --- |
| OsEp-Plm-15B10 | *Enterobacter sacchari* | 100.00 | 0.00 | **Fungicidal** |
| OsEp-Plm-30B1 | *Microbacterium testaceum* | 1.90 | 98.10 | Fungistatic |
| OsEp-Plm-15B6 | *Pantoea ananatis* | 37.14 | 62.86 | Fungistatic |
| OsEp-Plm-30B2 | *Pantoea ananatis* | 100.00 | 0.00 | **Fungicidal** |
| OsEp-Plm-30B6 | *Pantoea ananatis* | 100.00 | 0.00 | **Fungicidal** |
| OsEp-Plm-30B8 | *Pantoea ananatis* | 25.71 | 74.29 | Fungistatic |
| OsEp-Plm-30B17 | *Pantoea ananatis* | 100.00 | 0.00 | **Fungicidal** |
| OsEp-Plm-30B19 | *Pantoea ananatis* | 100.00 | 0.00 | **Fungicidal** |
| OsEp-Plm-15B14 | *Pantoea dispersa* | 25.71 | 74.29 | Fungistatic |
| OsEp-Plm-30B3 | *Pantoea vagans* | 100.00 | 0.00 | **Fungicidal** |
| OsEp-Plm-15B16 | *Pseudomonas oryzihabitans* | 20.00 | 80.00 | Fungistatic |
| OsEp-Plm-30B4 | *Rhizobium* sp. | 100.00 | 0.00 | **Fungicidal** |
|  | Mock | 0.00 | 100.00 |  |
|  | C.D. | 6.71 | 6.71 |  |
|  | SE(m) | 2.30 | 2.30 |  |
|  | SE(d) | 3.25 | 3.25 |  |
|  | C.V. (%) | 5.90 | 12.27 |  |
|  | F (calc.) | 315.57 | 315.57 |  |
|  | F (tab.) | 2.22 | 2.22 |  |

**Supplementary Table 7.** Effect of phyllosphere bacterial interactions on rice seed germination

| Bacterial isolates | **Seed germination (%)** | | | |
| --- | --- | --- | --- | --- |
|  | 10^6-7^ | 10^7-8^ | 10^8-9^ | 10^9-10^ |
| Mock | 98.7 | 98.7 | 98.7 | 0 |
| *Enterobacter sacchari*  OsEp-Plm-15B10 | 98.0 | 92.0 | 86.7 | 0 |
| *Microbacterium testaceum*  OsEp-Plm-30B1 | 98.7 | 86.7 | 82.0 | 0 |
| *Pantoea ananatis*  OsEp-Plm-15B6 | 96.0 | 90.0 | 80.7 | 0 |
| *Pantoea ananatis*  OsEp-Plm-30B17 | 95.3 | 88.7 | 78.7 | 0 |
| *Pantoea dispersa*  OsEp-Plm-15B14 | 92.0 | 90.0 | 82.0 | 0 |
| *Pantoea vagans*  OsEp-Plm-30B3 | 96.0 | 88.0 | 81.3 | 0 |
| *Pseudomonas oryzihabitans*  OsEp-Plm-15B16 | 98.7 | 89.3 | 82.7 | 0 |
| *Rhizobium* sp.  OsEp-Plm-30B4 | 96.7 | 90.0 | 79.3 | 0 |
| *Sphingomonas* sp.  OsEp-Plm-15B2 | 95.3 | 92.0 | 83.3 | 0 |
| Mean | 93.0 | 87.1 | 79.9 | 0 |
|  | Titer | Isolates | Interactions |  |
| F cal | 289.7 | 228.3 | 3.2 |  |
| F tab | 3.1 | 1.8 | 1.6 |  |

**Supplementary Table 8.** Shoot growth alteration on rice seedlings induced by phyllosphere bacterial isolates

| **Bacterial isolate** | **Bacterial Titer (CFU mL^-1^)** | | | | | | | | |
| --- | --- | --- | --- | --- | --- | --- | --- | --- | --- |
|  | **Shoot length (mm)** | | | | **Shoot length deviation (%)** | | | | |
|  | **10^6-7^** | **10^7-8^** | **10^8-9^** | **Mean** | **10^6-7^** | **10^7-8^** | **10^8-9^** | | **Mean** |
| ***Enterobacter sacchari***  **OsEp-Plm-15B10** | 34.4 | 28.2 | 10.8 | 24.5 | 1.2 | -17.1 | -68.2 | | -28.0 |
| ***Microbacterium testaceum***  **OsEp-Plm-30B1** | 32.0 | 27.4 | 20.6 | 26.7 | -5.9 | -19.4 | -39.4 | | -21.5 |
| ***Pantoea ananatis***  **OsEp-Plm-15B6** | 35.0 | 23.8 | 10.2 | 23.0 | 3.0 | -30.1 | -70.0 | | -32.3 |
| ***Pantoea ananatis***  **OsEp-Plm-30B17** | 30.6 | 22.2 | 11.4 | 21.4 | -10.0 | -34.7 | -66.4 | | -37.0 |
| ***Pantoea dispersa***  **OsEp-Plm-15B14** | 29.0 | 22.2 | 18.8 | 23.3 | -14.7 | -34.7 | -44.7 | | -31.4 |
| ***Pantoea vagans***  **OsEp-Plm-30B3** | 31.8 | 31.4 | 19.6 | 27.6 | -6.4 | -7.6 | -42.4 | | -18.8 |
| ***Pseudomonas oryzihabitans***  **OsEp-Plm-15B16** | 36.2 | 32.6 | 19.8 | 29.5 | 6.5 | -4.1 | -41.7 | | -13.1 |
| ***Rhizobium* sp.**  **OsEp-Plm-30B4** | 29.0 | 20.4 | 20.0 | 23.1 | -14.7 | -40.0 | -41.1 | | -31.9 |
| ***Sphingomonas* sp.**  **OsEp-Plm-15B2** | 32.0 | 30.8 | 22.2 | 28.3 | -5.8 | -9.4 | -34.7 | | -16.6 |
| **Mock** | **34.0** | **34.0** | **34.0** | **34.0** | **-** | **-** | **-** | | **-** |
| Mean | 32.2 | 27.0 | 17.8 |  | -5.6 | -22.0 | -51.3 | |  |
|  | Titer | Isolates | Interaction |  | Titer | Isolates | Interaction |  | |
| F cal | 1764.3 | 82.6 | 38.3 |  | 1681.3 | 40.4 | 26.7 | |  |
| F tab | 3.0 | 1.8 | 1.6 |  | 3.1 | 1.8 | 1.6 | |  |

**Supplementary Table 9**. Root growth alteration on rice seedlings induced by phyllosphere bacterial isolates

| **Bacterial isolate** | **Bacterial Titer** | | | | | | | |
| --- | --- | --- | --- | --- | --- | --- | --- | --- |
|  | **Root length (mm)** | | | | **Root length deviation (%)** | | | |
|  | **10^6-7^** | **10^7-8^** | **10^8-9^** | **Mean** | **10^6-7^** | **10^7-8^** | **10^8-9^** | **Mean** |
| ***Enterobacter sacchari***  **OsEp-Plm-15B10** | 32.8 | 12.6 | 2.8 | 16.1 | 5.0 | -59.4 | -91.0 | -48.5 |
| ***Microbacterium testaceum* OsEp-Plm-30B1** | 31.8 | 23.8 | 12.4 | 22.7 | 2.1 | -23.6 | -60.2 | -27.3 |
| ***Pantoea ananatis***  **OsEp-Plm-15B6** | 22.4 | 18.4 | 8.4 | 16.4 | -28.2 | -41.0 | -73.1 | -47.4 |
| ***Pantoea ananatis***  **OsEp-Plm-30B17** | 20.4 | 20.8 | 3.6 | 14.9 | -34.6 | -33.4 | -88.4 | -52.1 |
| ***Pantoea dispersa***  **OsEp-Plm-15B14** | 12.6 | 10.2 | 7.4 | 10.1 | -59.5 | -67.3 | -76.2 | -67.7 |
| ***Pantoea vagans***  **OsEp-Plm-30B3** | 31.6 | 21.6 | 11.6 | 21.6 | 1.4 | -30.6 | -62.7 | -30.6 |
| ***Pseudomonas oryzihabitans* OsEp-Plm-15B16** | 37.4 | 31.6 | 22.4 | 30.5 | 20.0 | 1.4 | -28.2 | -2.3 |
| ***Rhizobium* sp.**  **OsEp-Plm-30B4** | 31.8 | 20.6 | 8.6 | 20.3 | 2.1 | -33.9 | -72.2 | -34.7 |
| ***Sphingomonas* sp.**  **OsEp-Plm-15B2** | 31.8 | 23.6 | 11.4 | 22.3 | 2.1 | -24.3 | -63.4 | -28.5 |
| **Mock** | 31.2 | 31.2 | 31.2 | 31.2 | - | - | - | - |
| **Mean** | 29.2 | 22.9 | 12.0 |  | -6.9 | -28.6 | -66.1 |  |
|  | Titer | Isolates | Interaction |  | Titer | Isolates | Interaction |  |
| **F cal** | 1813.0 | 205.6 | 42.5 |  | 1705.8 | 155.5 | 29.9 |  |
| **F tab** | 3.0 | 1.8 | 1.6 |  | 3.1 | 1.8 | 1.6 |  |

**Supplementary Table 10.** qPCR analysis of transcriptional response of defense genes in phyllobacterized rice seedlings

| **Gene** | **Fold change** | | |
| --- | --- | --- | --- |
|  | **Hour Post Inoculation** | | |
|  | **24 h** | **48 h** | **72 h** |
| ***OsCEBiP*** |  |  |  |
| *Sphingomonas* sp. OsEp-Plm-15B2 | **1.60** | **1.88** | 0.76 |
| *Pantoea ananatis* OsEp-Plm-15B6 | **2.89** | **1.84** | **1.62** |
| *Enterobacter sacchari* OsEp-Plm-15B10 | **1.30** | **1.94** | **1.61** |
| *Pantoea dispersa* OsEp-Plm-15B14 | **2.11** | **4.06** | **2.23** |
| *Pseudomonas oryzihabitans* OsEp-Plm-15B16 | **1.78** | **3.16** | **2.35** |
| *Microbacterium testaceum* OsEp-Plm-30B1 | **2.26** | **3.10** | **4.50** |
| *Pantoea vagans* OsEp-Plm-30B3 | **2.00** | **2.74** | 1.05 |
| *Rhizobium* sp. OsEp-Plm-30B4 | **2.13** | **1.92** | **2.14** |
| *Pantoea ananatis* OsEp-Plm-30B17 | **1.56** | **3.36** | **2.66** |
| ***OsCERK1*** |  |  |  |
| *Sphingomonas* sp. OsEp-Plm-15B2 | **1.65** | **1.38** | 0.85 |
| *Pantoea ananatis* OsEp-Plm-15B6 | **1.53** | **1.26** | 0.99 |
| *Enterobacter sacchari* OsEp-Plm-15B10 | **1.78** | **1.29** | **1.15** |
| *Pantoea dispersa* OsEp-Plm-15B14 | **1.97** | **1.38** | **1.46** |
| *Pseudomonas oryzihabitans* OsEp-Plm-15B16 | **1.75** | **1.85** | **1.64** |
| *Microbacterium testaceum* OsEp-Plm-30B1 | **1.16** | **1.17** | **1.50** |
| *Pantoea vagans* OsEp-Plm-30B3 | **1.38** | **1.20** | 0.90 |
| *Rhizobium* sp. OsEp-Plm-30B4 | **1.40** | **1.16** | **2.31** |
| *Pantoea ananatis* OsEp-Plm-30B17 | **1.24** | 1.05 | 1.07 |
| ***OsPAD4*** |  |  |  |
| *Sphingomonas* sp. OsEp-Plm-15B2 | 1.06 | 1.07 | 0.79 |
| *Pantoea ananatis* OsEp-Plm-15B6 | 0.80 | **1.61** | 0.74 |
| *Enterobacter sacchari* OsEp-Plm-15B10 | **1.37** | **1.88** | 1.08 |
| *Pantoea dispersa* OsEp-Plm-15B14 | **1.15** | **2.16** | 0.95 |
| *Pseudomonas oryzihabitans* OsEp-Plm-15B16 | 0.81 | **1.82** | 0.98 |
| *Microbacterium testaceum* OsEp-Plm-30B1 | 0.87 | **1.20** | 0.80 |
| *Pantoea vagans* OsEp-Plm-30B3 | 0.87 | 1.05 | 0.61 |
| *Rhizobium* sp. OsEp-Plm-30B4 | 0.87 | **1.17** | 0.73 |
| *Pantoea ananatis* OsEp-Plm-30B17 | 0.86 | 1.07 | 0.76 |
| ***OsEDS1*** |  |  |  |
| *Sphingomonas* sp. OsEp-Plm-15B2 | **2.06** | 0.85 | 1.07 |
| *Pantoea ananatis* OsEp-Plm-15B6 | **1.51** | **1.76** | 0.62 |
| *Enterobacter sacchari* OsEp-Plm-15B10 | 0.92 | **1.25** | 1.07 |
| *Pantoea dispersa* OsEp-Plm-15B14 | **1.12** | **1.32** | **1.82** |
| *Pseudomonas oryzihabitans* OsEp-Plm-15B16 | **1.34** | 0.89 | **1.18** |
| *Microbacterium testaceum* OsEp-Plm-30B1 | **1.34** | 0.79 | 1.07 |
| *Pantoea vagans* OsEp-Plm-30B3 | **1.54** | 0.92 | 0.85 |
| *Rhizobium* sp. OsEp-Plm-30B4 | **1.64** | 0.76 | 0.72 |
| *Pantoea ananatis* OsEp-Plm-30B17 | **1.25** | 0.94 | **1.17** |
| ***OsPDF2.2*** |  |  |  |
| *Sphingomonas* sp. OsEp-Plm-15B2 | **1.86** | 0.90 | 1.00 |
| *Pantoea ananatis* OsEp-Plm-15B6 | **1.39** | 0.93 | 0.67 |
| *Enterobacter sacchari* OsEp-Plm-15B10 | **1.08** | **1.51** | 0.89 |
| *Pantoea dispersa* OsEp-Plm-15B14 | **1.20** | **1.14** | 0.84 |
| *Pseudomonas oryzihabitans* OsEp-Plm-15B16 | **1.75** | **1.88** | 0.76 |
| *Microbacterium testaceum* OsEp-Plm-30B1 | 0.98 | **1.79** | 0.97 |
| *Pantoea vagans* OsEp-Plm-30B3 | 1.07 | **2.06** | 0.95 |
| *Rhizobium* sp. OsEp-Plm-30B4 | 0.80 | **1.21** | **1.14** |
| *Pantoeaananatis* OsEp-Plm-30B17 | 1.07 | 0.99 | 1.00 |
| ***OsPR1.1*** |  |  |  |
| *Sphingomonas* sp. OsEp-Plm-15B2 | **1.30** | **2.39** | 0.66 |
| *Pantoea ananatis* OsEp-Plm-15B6 | **1.34** | **1.45** | **1.21** |
| *Enterobacter sacchari* OsEp-Plm-15B10 | **1.68** | **3.06** | 1.04 |
| *Pantoea dispersa* OsEp-Plm-15B14 | **1.56** | **1.76** | **1.88** |
| *Pseudomonas oryzihabitans* OsEp-Plm-15B16 | **1.13** | **1.59** | 0.93 |
| *Microbacterium testaceum* OsEp-Plm-30B1 | **1.13** | **1.20** | 0.70 |
| *Pantoea vagans* OsEp-Plm-30B3 | **1.14** | 0.96 | 0.76 |
| *Rhizobium* sp. OsEp-Plm-30B4 | **1.54** | 0.93 | 1.04 |
| *Pantoea ananatis* OsEp-Plm-30B17 | **1.19** | **1.61** | 1.02 |
| ***OsFMO*** |  |  |  |
| *Sphingomonas* sp. OsEp-Plm-15B2 | **1.23** | 0.80 | **1.28** |
| *Pantoea ananatis* OsEp-Plm-15B6 | **1.48** | **1.35** | 0.87 |
| *Enterobacter sacchari* OsEp-Plm-15B10 | 1.09 | **1.60** | **1.13** |
| *Pantoea dispersa* OsEp-Plm-15B14 | **1.83** | **1.31** | **1.29** |
| *Pseudomonas oryzihabitans* OsEp-Plm-15B16 | 0.85 | 0.65 | 1.03 |
| *Microbacterium testaceum* OsEp-Plm-30B1 | 0.92 | 0.75 | **1.11** |
| *Pantoea vagans* OsEp-Plm-30B3 | **1.53** | **1.40** | 0.96 |
| *Rhizobium* sp. OsEp-Plm-30B4 | **1.30** | 0.91 | 1.05 |
| *Pantoea ananatis* OsEp-Plm-30B17 | **1.66** | 1.04 | 0.90 |
| ***OsNPR1*** |  |  |  |
| *Sphingomonas* sp. OsEp-Plm-15B2 | 0.83 | 0.84 | 1.08 |
| *Pantoea ananatis* OsEp-Plm-15B6 | **1.28** | **1.27** | 1.04 |
| *Enterobacter sacchari* OsEp-Plm-15B10 | 0.90 | 1.02 | 1.12 |
| *Pantoea dispersa* OsEp-Plm-15B14 | 1.07 | 1.02 | **1.14** |
| *Pseudomonas oryzihabitans* OsEp-Plm-15B16 | 0.70 | 1.05 | **1.15** |
| *Microbacterium testaceum* OsEp-Plm-30B1 | **1.21** | 0.77 | 0.95 |
| *Pantoea vagans* OsEp-Plm-30B3 | 0.90 | 0.79 | 1.09 |
| *Rhizobium* sp. OsEp-Plm-30B4 | 0.84 | 0.96 | **1.16** |
| *Pantoea ananatis* OsEp-Plm-30B17 | 1.00 | 0.95 | **1.30** |

***Bold= Up- regulated; Red font= Significant up-regulation**
